# Medicaid Eligibility Loss Among Dual-Eligible Beneficiaries Before and During COVID-19 Public Health Emergency

**DOI:** 10.1001/jamanetworkopen.2024.5876

**Published:** 2024-04-11

**Authors:** Yanlei Ma, Eric T. Roberts, Kenton J. Johnston, E. John Orav, Jose F. Figueroa

**Affiliations:** 1 Department of Health Policy & Management, Harvard T.H. Chan School of Public Health, Boston, Massachusetts; 2 University of Pennsylvania, Philadelphia; 3 Washington University in St Louis, St Louis, Missouri; 4 Department of Medicine, Brigham and Women’s Hospital, Harvard Medical School, Boston, Massachusetts

## Abstract

**Importance:**

Medicaid coverage loss can substantially compromise access to and affordability of health care for dual-eligible beneficiaries. The extent to which this population lost Medicaid coverage before and during the COVID-19 public health emergency (PHE) and the characteristics of beneficiaries more at risk for coverage loss are currently not well known.

**Objective:**

To assess the loss of Medicaid coverage among dual-eligible beneficiaries before and during the first year of the PHE, and to examine beneficiary-level and plan-level factors associated with heightened likelihood of losing Medicaid.

**Design, Setting, and Participants:**

This repeated cross-sectional study used national Medicare data to estimate annual rates of Medicaid loss among dual-eligible beneficiaries before (2015 to 2019) and during the PHE (2020). Individuals who were dual eligible for Medicare and Medicaid at the beginning of a given year and who continuously received low-income subsidies for Medicare Part D prescription drug coverage were included in the sample. Multivariable regression models were used to examine beneficiary-level and plan-level factors associated with Medicaid loss. Data analyses were conducted between March 2023 and October 2023.

**Exposure:**

Onset of PHE.

**Main Outcomes and Measures:**

Loss of Medicaid for at least 1 month within a year.

**Results:**

Sample included 56 172 736 dual-eligible beneficiary-years between 2015 and 2020. In 2020, most dual-eligible beneficiaries were aged over 65 years (5 984 420 [61.1%]), female (5 868 866 [59.9%]), non-Hispanic White (4 928 035 [50.3%]), full-benefit eligible (6 837 815 [69.8%]), and enrolled in traditional Medicare (5 343 537 [54.6%]). The adjusted proportion of dual-eligible beneficiaries losing Medicaid for at least 1 month increased from 6.6% in 2015 to 7.3% in 2019 and then dropped to 2.3% in 2020. Between 2015 and 2019, dual-eligible beneficiaries who were older (ages 55-64 years: −1.4%; 95% CI, −1.8% to −1.0%; ages 65-74 years: −2.0%; 95% CI, −2.5% to −1.5%; ages 75 and older: −4.5%; 95% CI, −5.0% to −4.0%), disabled (−0.8%; 95% CI, −1.1% to −0.6%), and in integrated care programs were less likely to lose Medicaid. In 2020, the disparities within each of these demographic groups narrowed significantly. Notably, while Black (0.6%; 95% CI, 0.2% to 0.9%) and Hispanic (0.7%; 95% CI, 0.3% to 1.2%) dual-eligible beneficiaries were more likely to lose Medicaid than their non-Hispanic White counterparts between 2015 and 2019, such gap was eliminated for Black beneficiaries and narrowed for Hispanic beneficiaries in 2020.

**Conclusions and Relevance:**

During the PHE, Medicaid coverage loss declined significantly among dual-eligible beneficiaries, and disparities were mitigated across subgroups. As the PHE unwinds, it is crucial for policymakers to implement strategies to minimize Medicaid coverage disruptions and racial and ethnic disparities, especially given that loss of Medicaid was slightly increasing over time before the PHE.

## Key Points

**Question:**

To what extent did dual-eligible beneficiaries lose Medicaid before and during the COVID-19 public health emergency (PHE), and what characteristics of beneficiaries were associated with increased risk of Medicaid loss?

**Findings:**

In this repeated cross-sectional study, dual-eligible beneficiaries losing Medicaid increased steadily prior to the PHE and fell significantly during the PHE. Beneficiaries who were older, disabled, eligible for full Medicaid benefits, and in integrated care programs were less likely to lose coverage; racial disparities in coverage loss observed prior to PHE were largely eliminated during the PHE.

**Meaning:**

The PHE Medicaid continuous enrollment provision was associated with reduced Medicaid loss and disparities among dual-eligible beneficiaries.

## Teaser

This repeated cross-sectional study examines rates of Medicaid coverage loss among dual-eligible beneficiaries and characteristics associated with coverage loss during the COVID-19 public health emergency.

## Introduction

Approximately 12.5 million people in the US are dually eligible for the Medicare and Medicaid programs.^1^ Although dual-eligible beneficiaries typically remain continuously enrolled in Medicare, many face the risk of periodic Medicaid coverage loss due to various factors including income fluctuations, state-specific changes in Medicaid eligibility, and most notably, administrative barriers associated with Medicaid redeterminations.^2,3,4^ These redeterminations, which states perform at least annually, require dual-eligible beneficiaries to provide detailed documentation of their income and assets.^5^ One concern is that beneficiaries who have difficulty navigating this redetermination process may lose Medicaid even if they remain eligible. Because dual-eligible beneficiaries have complex medical and social needs,^6,7^ the loss of Medicaid can leave them without access to vital health care services.^6,8^ This is because Medicaid provides substantial financial assistance to cover Medicare copays, deductibles, and premiums,^6^ and also covers services such as long-term care, behavioral health, and dental care that are not covered by Medicare.^6^ The loss of Medicaid may disproportionately affect certain subpopulations of this population, including individuals from racial and ethnic minority groups who may encounter structural discrimination and language or cultural barriers, as well as those with cognitive or intellectual disabilities.^2,8,9,10^

During the COVID-19 public health emergency (PHE), states paused Medicaid eligibility redeterminations, enabling dual-eligible beneficiaries to remain continuously enrolled in Medicaid without having to verify continued eligibility. However, with the official conclusion of the PHE and termination of the Medicaid continuous enrollment provision,^11^ states have resumed Medicaid redeterminations as of April 1, 2023.^12^ Consequently, dual-eligible beneficiaries once again face the risk of losing Medicaid. Understanding the extent to which dual-eligible beneficiaries may again lose Medicaid coverage, and factors associated with the risk of coverage loss, is important for identifying subgroups of this population who may need additional assistance during the redetermination process.

While some studies have explored factors associated with Medicaid loss among dual-eligible beneficiaries,^2,3,4^ we are unaware of any study that has assessed Medicaid disenrollment in this population before and during the PHE. Moreover, prior studies did not thoroughly examine factors associated with Medicaid loss, such as race and ethnicity, dual-eligible status, and Medicare coverage type, including traditional fee-for-service Medicare (TM), conventional Medicare Advantage (MA) plans, and Medicare managed care models that integrate Medicare and Medicaid benefits.^13^

Therefore, using national Medicare data from 2015 to 2020, we addressed the following questions. First, to what extent did dual-eligible beneficiaries lose Medicaid prior to the PHE? Second, was the PHE and pause on Medicaid redeterminations associated with changes in Medicaid loss during the first year of the pandemic? Third, what patient- and plan-level characteristics were associated with Medicaid loss among dual-eligible beneficiaries?

## Methods

This study was approved by the Harvard School of Public Health institutional review board and the requirement for informed consent was waived because data were deidentified. The reporting of results in this study followed the Strengthening the Reporting of Observational Studies in Epidemiology (STROBE) reporting guidelines.

### Data

We used 100% administrative data from the Medicare Beneficiary Summary Files (MBSF) between 2015 and 2020, which contain information on beneficiaries’ demographics, monthly dual-eligibility status, enrollment in TM vs MA, and eligibility for subsidized Part D benefits. We linked the MBSF to the Medicare plan characteristics files, which allowed us to determine dual-eligible beneficiaries’ enrollment in Program of All-Inclusive Care for the Elderly (PACE), state-specific Medicare-Medicaid plans (MMPs), fully integrated dual-eligible special needs plans (FIDE SNPs), other dual-eligible special needs plans (other D-SNPs) as well as other conventional, nonintegrated MA plans. Similar to a prior study, we excluded Texas from the analyses in 2015 due to an implausibly high proportion (96.4%) of dual-eligible beneficiaries losing Medicaid coverage in that year.^4^

### Statistical Analyses

We conducted a repeated cross-sectional study of dual-eligible beneficiaries between 2015 and 2020. In each year, among beneficiaries who were dually eligible for Medicaid in January of the year, we identified those who lost Medicaid coverage entirely (ie, not qualifying for any Medicaid benefits) for at least 1 month, 3 months, and 6 months, respectively. To focus on beneficiaries who likely disenrolled due to administrative barriers (ie, disenrolled despite likely remaining eligible for Medicaid), we limited the sample to those who continuously received Part D low-income subsidies throughout the study year. This is because the eligibility requirements for Part D low-income subsidies are similar to those of Medicaid, and almost all beneficiaries who qualify for a full Part D subsidy qualify for full or partial Medicaid coverage. Additionally, beneficiaries who died within a given year were excluded from that year’s analyses.

Next, we calculated annual unadjusted and adjusted rate of Medicaid disenrollment among dual-eligibles, including both the overall disenrollment rate and the disenrollment rates stratified by dual-eligibility status, age group, gender, race and ethnicity, original reason for Medicare entitlement, as well as TM and MA plan type. Race and ethnicity were defined using the Research Triangle Institute race code variable,^14^ and were analyzed given the known administrative barriers of maintaining Medicaid coverage for people of color.^15,16^ To estimate the unadjusted rate of Medicaid disenrollment in each year, among those dually eligible in January of that year, we calculated the proportion of beneficiaries who had completely lost their Medicaid coverage (ie, not qualifying for any Medicaid benefits) for at least 1 month at any point during the remainder of that year.

To estimate the adjusted rate of Medicaid disenrollment in each year, we estimated a beneficiary-year–level logistic regression model using pooled data between 2015 and 2020. The dependent variable of the regression was whether a beneficiary had completely lost Medicaid for at least 1 month in a given year, and the explanatory variables of the regression included the beneficiary’s demographic characteristics, TM and MA plan type, the interactions between each covariate and the full set of year indicators, and county fixed effects. The interaction terms allowed the relationship between the disenrollment pattern and beneficiary-level characteristics to vary across years, hence capturing any potential change in disenrollment pattern due to the PHE. The county fixed effects were included to account for differences in availability of integrated care plans across counties and differences in state policies that may influence the ease of Medicaid redetermination.^2,3^ The standard errors were clustered at the state level to address any unmeasured differences in state-level Medicaid policy environments that may affect Medicaid redetermination.^2,3^ The adjusted annual rates were then calculated as the average estimated likelihood of dual-eligible beneficiaries losing Medicaid coverage in each year.

Finally, to understand how disenrollment rates differ by beneficiary- and plan-level characteristics and how such difference varied before vs during the PHE, we reported the average marginal effects (ie, the average relative likelihood of losing Medicaid coverage compared with the reference group) and their 95% confidence intervals for each demographic and plan-level characteristic for 2015-2019 and 2020, respectively.

#### Sensitivity Analyses

In sensitivity analyses, we calculated the annual unadjusted rate of Medicaid disenrollment for all dual-eligible beneficiaries irrespective of whether they received Part D low-income subsidies. Additionally, we reestimated the average marginal effects for Medicaid disenrollment among dual-eligible beneficiaries using April to December data as Medicaid continuous enrollment provision took effect after the March 2020 Families First Coronavirus Response Act. Using a 2018 sample available to our research group, we also reestimated the average marginal effects for Medicaid disenrollment among dual-eligible beneficiaries, including beneficiaries’ hierarchical condition categories (HCC) scores as an additional explanatory variable because the health conditions of dual-eligible beneficiaries may impact their likelihood of disenrolling from Medicaid and Medicare choice. Finally, we reestimated the average marginal effects for Medicaid disenrollment without clustering standard errors at the state level.

The threshold for statistical significance was *P* < .05 using 2-sided tests. Analyses were performed using R version 4.2.1 (R Project for Statistical Computing).

## Results

### Characteristics of Dual-Eligible Beneficiaries

Our sample included 56 172 736 dual-eligible beneficiary-years between 2015 to 2020. In 2020, the majority of the dual-eligible beneficiaries were aged over 65 years (5 984 420 [61.1%]), female (5 868 866 [59.9%]), non-Hispanic White (4 928 035 [50.3%]), full-benefit eligible (6 837 815 [69.8%]), and enrolled in TM (5 343 537 [54.6%]). Characteristics of dual-eligible beneficiaries in 2015 were similar to those in 2020 except that more dual-eligible beneficiaries were enrolled in D-SNPs in 2020 (24.3% in 2020 vs 14.1% in 2018) (eTable in Supplement 1).

### Share of Medicaid Coverage Loss Among Dual-Eligibles

**Figure 1.**
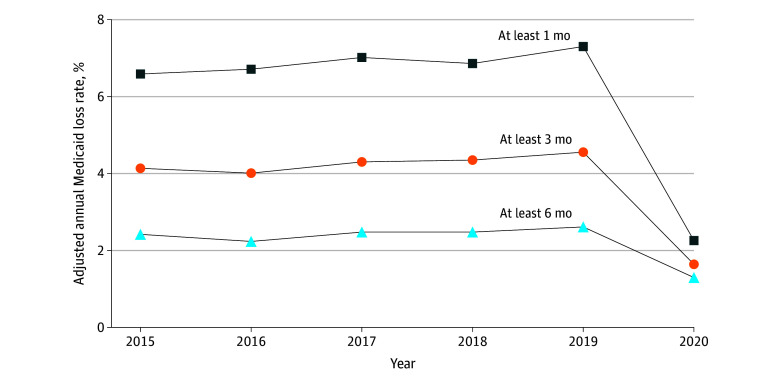
Adjusted Annual Medicaid Coverage Loss Rate, All Dual-Eligible Beneficiaries, 2015-2020 Sample limited to beneficiaries who were dually eligible for Medicaid as of January of the year and continuously received low-income subsidies for Medicare Part D prescription drug coverage throughout the year. Beneficiaries who died within a year were excluded from the analyses. Adjusted annual Medicaid coverage loss rate represents the proportion of dual-eligible beneficiaries in the sample who lost Medicaid coverage entirely (ie, not qualifying for any Medicaid benefits) in a given year after accounting for beneficiary’s age, gender, race and ethnicity, dual-eligible status, original reason for Medicare entitlement, traditional fee-for-service and Medicare Advantage plan type, and county fixed effects.

The adjusted share of dual-eligible beneficiaries experiencing Medicaid loss for at least 1 month rose from 6.6% in 2015 to 7.3% in 2019, before dropping to 2.3% in 2020 (Figure 1). Similar patterns were found in the adjusted rates of Medicaid loss for at least 3 months and 6 months, respectively (Figure 1). Among dual-eligible beneficiaries who lost Medicaid coverage for at least 1 month in a given year, an average of 53.7% regained their Medicaid coverage within 12 months (eFigure 1 in Supplement 1).

**Figure 2.**
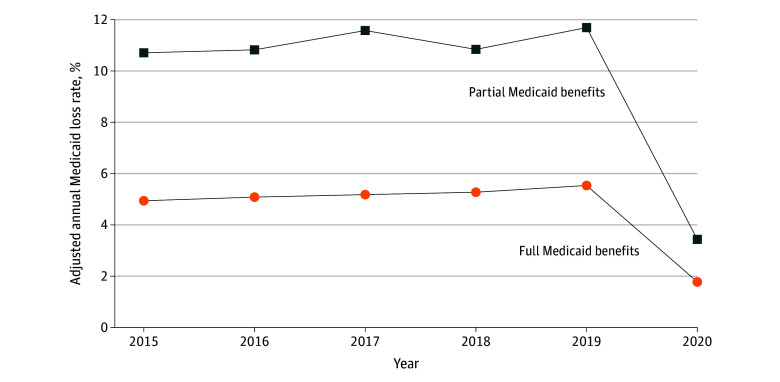
Adjusted Annual Medicaid Coverage Loss Rate for At Least 1 Month, By Dual-Eligible Status, 2015-2020 Sample limited to beneficiaries who were dually eligible for Medicaid as of January of each year and continuously received Part D low-income subsidies throughout the year. Beneficiaries who died within a year were excluded from the analyses. Adjusted annual Medicaid coverage loss rate represents the proportion of dual-eligibles in the sample who lost Medicaid coverage entirely (ie, not qualifying for any Medicaid benefits) in a given year after accounting for beneficiary’s age, gender, race and ethnicity, dual-eligible status, original reason for Medicare entitlement, traditional fee-for-service and Medicare Advantage plan type, and county fixed effects. Full Medicaid benefits dual-eligible beneficiaries include qualified Medicare beneficiary (QMB) with full Medicaid coverage, specified low-income Medicare beneficiary (SLMB) with full Medicaid coverage, and other dual-eligible beneficiaries with full Medicaid coverage. Partial Medicaid benefits dual-eligible beneficiaries include QMB-only, SLMB-only, qualified disabled working individual, and qualifying individuals.

Dual-eligible beneficiaries who had full Medicaid benefits at the beginning of the study years were less likely to lose Medicaid compared with those who had partial Medicaid benefits (Figure 2). Adjusted analyses show that between 2015 and 2019, an average of 5.2% of full-benefit dual-eligible beneficiaries lost Medicaid for at least 1 month, whereas 11.1% of partial-benefit dual-eligible beneficiaries lost Medicaid. This difference in disenrollment narrowed in 2020, with approximately 1.8% of full Medicaid benefit dual-eligible beneficiaries and 3.4% partial Medicaid benefit dual-eligible beneficiaries losing Medicaid for at least 1 month (Figure 2). Similar to the disenrollment pattern observed for dual-eligible beneficiaries with different Medicaid eligibility status, we also found disparities in Medicaid loss narrowed substantially in 2020 relative to prior years among different age, gender, race and ethnicity, and Medicare entitlement groups (eFigures 2-5 in Supplement 1).

**Figure 3.**
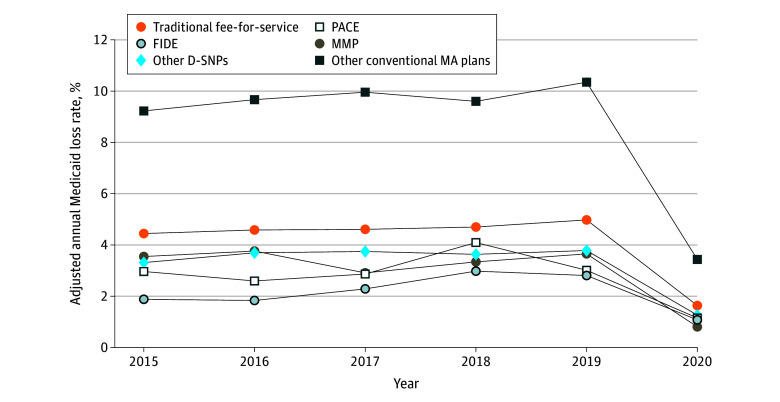
Adjusted Annual Medicaid Coverage Loss Rate for at Least 1 Month, by TM and MA Plan Type, 2015-2020 D-SNP indicates dual-eligible special needs plan; FIDE, fully integrated dual-eligible plan; MA, Medicare Advantage; PACE, Program for All-Inclusive Care for the Elderly; TM, traditional fee-for-service Medicare. Sample limited to beneficiaries who were dually eligible for Medicaid as of January of each year and continuously received Part D low-income subsidies throughout the year. Beneficiaries who died within a year were excluded from the analyses. For comparability across different TM and MA plan types, sample was further limited to dual-eligible beneficiaries aged 55 years or older with full Medicaid benefits. Adjusted annual Medicaid coverage loss rate represents the proportion of dual-eligible beneficiaries in the sample who lost Medicaid coverage entirely (ie, not qualifying for any Medicaid benefits) in a given year after accounting for beneficiary’s age, gender, race and ethnicity, dual-eligible status, original reason for Medicare entitlement, TM and MA plan type, and county fixed effects. Analysis includes beneficiaries in traditional Medicare and beneficiaries in MA plans with Part D. Beneficiaries in stand-alone drug plans, employer plans, cost plans, Medicare Savings Account plans, chronic condition special needs plans, or institutional special needs plans are excluded from analyses.

Dual-eligible beneficiaries enrolled in integrated care programs were less likely to lose Medicaid compared with those enrolled in TM or conventional, nonintegrated MA plans (Figure 3). Between 2015 and 2019, among full-benefit dual-eligible beneficiaries aged 55 years or older, the average adjusted rate of losing Medicaid for at least 1 month was 3.1% for those in PACE, 3.4% in MMPs, 2.4% in FIDE SNPs, and 3.6% in other D-SNPs. This contrasts with an average adjusted rate of 4.7% in TM and 9.8% in conventional, nonintegrated MA plans. In 2020, there was a notable reduction in Medicaid disenrollment across all TM and MA plan types. Among full-benefit dual-eligible beneficiaries aged 55 years or older, the average adjusted rates for losing Medicaid coverage for at least 1 month was 1.1% in PACE, 0.8% in MMPs, 1.1% in FIDE SNPs, and 1.2% in other D-SNPs, compared with 1.6% in TM and 3.4% in conventional, nonintegrated MA plans. Unadjusted rates demonstrated similar trends (eFigures 6-12 in Supplement 1).

### Relative Likelihood of Medicaid Coverage Loss Among Dual-Eligible Beneficiaries

**Figure 4.**
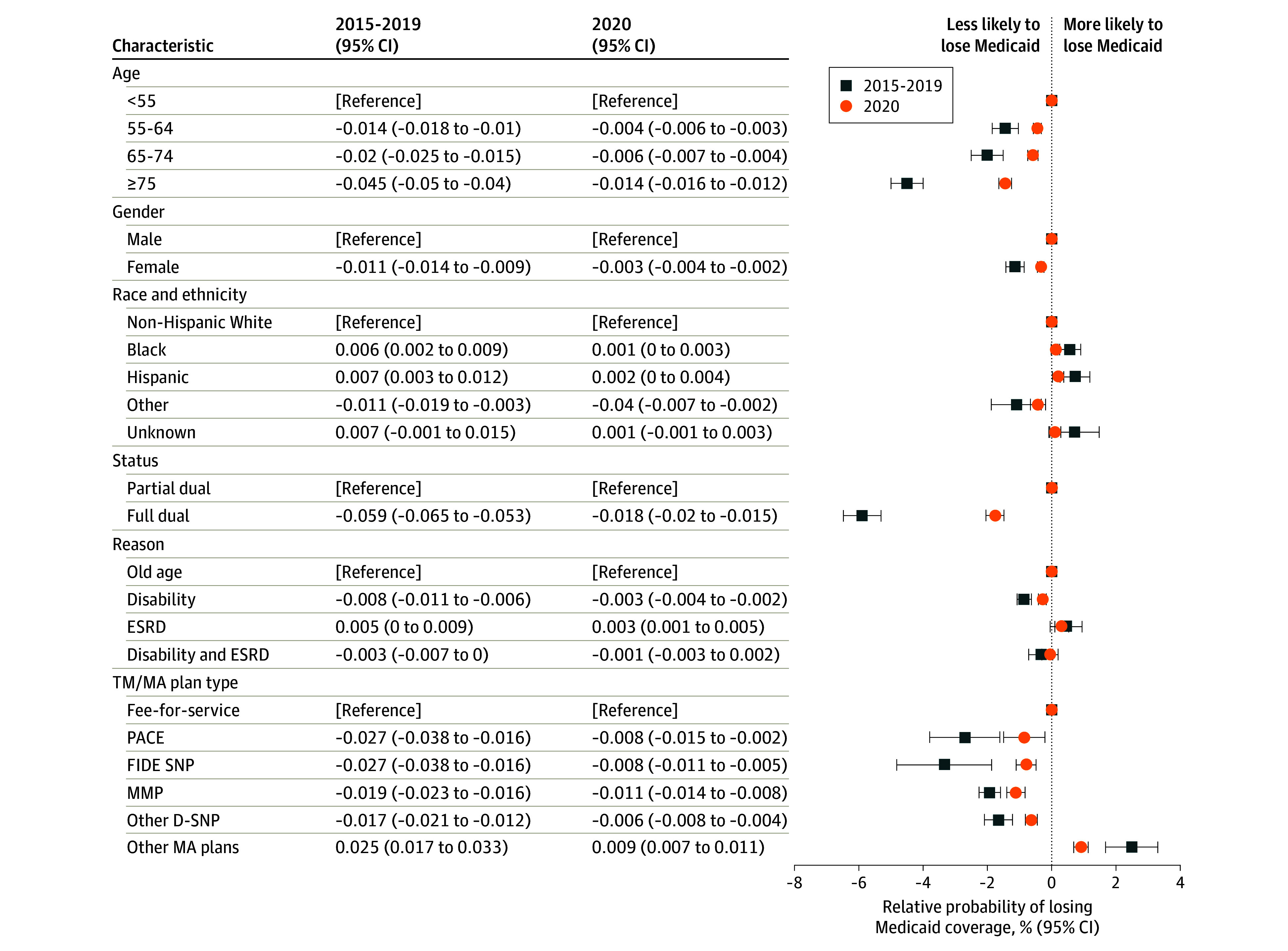
Relative Likelihood of Dual-Eligible Beneficiaries Losing Medicaid Coverage for at Least 1 Month, 2015-2019 vs 2020 D-SNP indicates dual-eligible special needs plan; ESRD, end-stage renal disease; FIDE, fully integrated dual-eligible plan; MA, Medicare Advantage; MMP, state-specific Medicare-Medicaid plans; PACE, Program of All-Inclusive Care for the Elderly; TM, traditional fee-for-service Medicare. Sample limited to beneficiaries who were dually eligible for Medicaid as of January of each year and continuously received low-income subsidies for Medicare Part D coverage throughout the year. Beneficiaries who died within a year were excluded from the analyses. Analysis includes beneficiaries in traditional Medicare and beneficiaries in MA plans with Part D. Beneficiaries in stand-alone drug plans, employer plans, cost plans, Medicare Savings Account plans, chronic condition special needs plans, or institutional special needs plans are excluded from analyses. Relative likelihood of dual-eligible beneficiaries losing Medicaid coverage is estimated from logistic regression model accounting for beneficiary’s age, gender, race and ethnicity, dual-eligible status, original reason for Medicare entitlement, TM and MA plan type, and county fixed effects. It represents the average difference in probability of losing Medicaid coverage compared with the reference group. Race and ethnicity were defined using the Research Triangle Institute race code. Other race and ethnicity includes Asian and Pacific Islander, American Indian and Alaska native, as well as any other race or ethnicity categories that are not non-Hispanic White, Black, or Hispanic.

Between 2015 and 2019, relative to those younger than age 55 years, the likelihood of dual-eligible beneficiaries losing Medicaid was lower among those aged 55 to 64 years (−1.4%; 95% CI, −1.8% to −1.0%), 65 to 74 years (−2.0%; 95% CI, −2.5% to −1.5%), and 75 years or older (−4.5%; 95% CI, −5.0% to −4.0%) (Figure 4). In addition, female beneficiaries were 1.1% less likely (95% CI, −1.4% to −0.9%) to disenroll from Medicaid than male; full-benefit dual-eligible beneficiaries were 5.9% less likely (95% CI, −6.5% to −5.3%) to disenroll than partial-benefit dual-eligible beneficiaries; and dual-eligible beneficiaries with disabilities were 0.8% less likely (95% CI, −1.1% to −0.6%) to disenroll than dual-eligible beneficiaries originally qualified for Medicare due to old age. Moreover, relative to their non-Hispanic White peers, Black beneficiaries were 0.6% (95% CI, 0.2% to 0.9%) more likely to lose Medicaid, and Hispanic beneficiaries were 0.7% (95% CI, 0.3% to 1.2%) more likely to lose Medicaid. Compared with dual-eligible beneficiaries enrolled in TM, the likelihood of losing Medicaid was lower for PACE (−2.7%; 95% CI, −3.8% to −1.6%), FIDE SNPs (−3.3%; 95% CI, −4.8% to −1.9%), MMPs (−1.9%; 95% CI, −2.3% to −1.6%), and other D-SNPs (−1.7%; 95% CI, −2.1% to −1.2%). Conversely, enrollees in conventional, nonintegrated MA plans were more likely to lose Medicaid than enrollees in TM (2.5%; 95% CI, 1.7% to 3.3%).

In 2020, while dual-eligible beneficiaries who were older, female, eligible for full Medicaid benefits, with disabilities, and enrolled in integrated care programs remain less likely to lose Medicaid, the disparities within each of these demographic groups narrowed significantly compared with 2015 to 2019. Moreover, in 2020, the likelihood of Black beneficiaries losing Medicaid was not significantly different from White beneficiaries (0.1%; 95% CI, −0.01% to 0.3%), although Hispanic beneficiaries were still 0.2% more likely (95% CI, 0.02% to 0.4%) to lose Medicaid than White beneficiaries.

### Sensitivity Analyses

Our findings remained qualitatively unchanged in sensitivity analyses. Specifically, our results were consistent with our main findings in models where we expanded the sample to include all dual-eligible beneficiaries regardless of whether they received the Part D low-income subsidy (eFigure 13 in Supplement 1), used only data between April and December each year (eFigure 14 in Supplement 1), incorporated HCC risk scores using 2018 data (eFigure 15 in Supplement 1), and did not cluster standard errors at the state level (eFigure 16 in Supplement 1).

## Discussion

From 2015 to 2019, the adjusted proportion of dual-eligible beneficiaries who lost Medicaid for at least 1 month rose from 6.6% to 7.3%. Over this period, Black and Hispanic beneficiaries were more likely to lose Medicaid than their non-Hispanic White peers. Conversely, dual-eligible beneficiaries who were older, female, eligible for full Medicaid, with disabilities, and enrolled in integrated care programs were less likely to lose Medicaid. In 2020, after the implementation of the continuous Medicaid eligibility provisions during the PHE, the adjusted proportion of dual-eligible beneficiaries who lost Medicaid for at least 1 month dropped to 2.3%. The reduction in Medicaid loss was consistent across demographic groups as well as TM and MA plan types. Notably, disparities in disenrollment rates between non-Hispanic White and Black beneficiaries was eliminated in 2020, and narrowed slightly between non-Hispanic White and Hispanic beneficiaries.

Our study sheds light on the challenges that dual-eligible beneficiaries faced in maintaining continuous Medicaid coverage prior to the PHE, and the challenges that will likely reemerge with the resumption of Medicaid redeterminations in 2023. Notably, we identified Medicaid coverage losses among dual-eligible beneficiaries who were qualified for Part D low-income subsidies, among whom Medicaid loss more likely reflected administrative barriers (eg, difficulty completing a redetermination) rather than a change in eligibility. A prior study suggested that administrative barriers to Medicaid renewal were the primary reason for disenrollment among full-benefit dual-eligible beneficiaries,^2^ while another analysis found that over 50% of dual-eligible beneficiaries who lost Medicaid continued to receive low-income subsidies, suggesting ongoing eligibility for Medicaid.^3^ Consistent with prior evidence,^3^ we found more than half of dual-eligible beneficiaries who lost Medicaid coverage subsequently regained their Medicaid coverage within 1 year. This suggests that the coverage losses are less likely due to sustained changes in eligibility than administrative factors. While the comprehensive redetermination processes allow states to manage and potentially limit the size and cost of their Medicaid populations, eligible individuals might lose Medicaid if they fail to receive, comprehend, or respond timely to notices or forms requesting additional information.^17,18^ Past research has found that states with more streamlined policies see lower disenrollment rates, making the process less daunting for Medicaid beneficiaries.^3^ As states reevaluate Medicaid eligibility for all enrollees—a process they are required to complete by May 2024—there is concern that the speed and scale of the redetermination process, coupled with these administrative hurdles, could result in unwarranted interruptions in Medicaid coverage.

The administrative barriers mentioned above can disproportionately affect people of color and others who face more structural discrimination and societal barriers. Our results show that Black and Hispanic beneficiaries were more likely to lose Medicaid compared with White beneficiaries prior to the PHE. In line with our findings, existing studies also show that individuals with limited English proficiency face higher risks of losing Medicaid during the renewal process compared with their English-proficient counterparts.^15,16^ Given that people of color are more often limited in English proficiency compared with White individuals, this challenge can exacerbate existing racial and ethnic enrollment disparities.

Our findings also suggest that certain Medicare plans may protect dual-eligible beneficiaries against the Medicaid coverage loss. Relative to dual-eligible beneficiaries enrolled in TM, those enrolled in integrated care programs—including both financially integrated care models (PACE, MMPs, and FIDE SNPs) and coordination-only D-SNPs—were less likely to experience Medicaid coverage loss. Conversely, those enrolled in other nonintegrated MA plans faced a higher risk of Medicaid loss. Such findings remained consistent in our sensitivity analyses after accounting for beneficiaries’ clinical risks. This aligns with previous research by Riley and colleagues^4^ that reported similar findings after adjusting for beneficiaries’ health risks and Medicare expenditure. One plausible explanation for such findings is that integrated care models have stronger incentives to keep their beneficiaries enrolled in Medicaid, as beneficiaries are required to disenroll if they lose Medicaid. In addition, states have collaborated with integrated care programs over the years to prevent unnecessary loss of Medicaid eligibility, such as mandating D-SNPs to assist their enrollees with Medicaid eligibility redetermination processes.^13^ Although our results suggest that there may be benefits of providing such assistance to D-SNP enrollees, to date, less than half of dual-eligible beneficiaries are enrolled in D-SNPs and other integrated care programs.^19^ Therefore, additional interventions are needed to protect dual-eligible beneficiaries in Medicare plans from losing Medicaid.

The substantial decline we observed in the disenrollment rate among dual-eligible beneficiaries in 2020 is likely due to the continuous enrollment provision during the PHE, which prevented states from redetermining beneficiaries’ eligibility for Medicaid. As the PHE ended, states resumed Medicaid redeterminations starting April 2023. Estimates suggest that over 15 million people could lose Medicaid, and recent evidence suggests that disenrollments overwhelmingly occur for procedural reasons rather than a change in eligibility.^10,20^ While current national estimates do not report Medicaid loss specifically among dual-eligible beneficiaries, our study underscores the potential for vulnerable groups, including people of color and those with disabilities, to experience disruptions in Medicaid coverage.^10,21,22^

Our results stress the importance for policymakers to take actions and mitigate Medicaid losses, particularly those due to administrative barriers. For example, states can adopt strategies to minimize Medicaid losses due to administrative barriers, including expanding the use of ex parte redeterminations, wherein enrollment and eligibility data from other means-tested programs, such as the Supplemental Nutrition Assistance Program, are used to automatically verify Medicaid eligibility.^23^ Other changes, including allowing enrollees an extended time window to respond to additional information requests, allocating more resources and personnel to process the volume of eligibility redeterminations, and providing accessible enrollment materials for beneficiaries with limited English proficiency or disabilities (eg, vision impairment),^24^ could also help to reduce administrative barriers to redetermination.

### Limitations

This study has several limitations. First, Medicaid disenrollment was estimated based on repeated cross-sectional analyses rather than following a cohort over an extended period. Therefore, we did not examine the extent to which the same individuals lost Medicaid across multiple years. Second, our analyses were based on beneficiary information in the MBSF, which does not capture all relevant factors that could influence disenrollment. Furthermore, unmeasured differences in the characteristics of dual-eligible beneficiaries across TM and MA plan types could have biased our estimates. Although we found similar associations between Medicare plan and Medicaid disenrollment in analyses of 2018 data that adjusted for Medicare HCC scores, a concern remains that unmeasured factors linked to Medicaid eligibility, such as the need for long-term nursing home care, could have differed across TM and MA plan types and biased our estimates. Third, our analyses excluded beneficiaries who died within a given year from the analyses of that year. Given that mortality rates may increase following the loss of Medicaid coverage for dual-eligible beneficiaries, our estimated rates of Medicaid coverage loss could be understated. Moreover, the mortality surge during the PHE could lead to conservative estimates of the reduction in Medicaid coverage loss associated with the continuous enrollment provision. Additionally, to the extent certain dual-eligible beneficiaries were more prone to mortality during the PHE, such selection might limit the generalizability of our findings. Fourth, beyond the continuous enrollment provision during the PHE, there might be other policy factors that may have influenced our findings.

## Conclusions

In this repeated cross-sectional study, we have documented recent trends in Medicaid coverage loss among dual-eligible beneficiaries, including in the wake of COVID-19 PHE. Our findings highlight the potential vulnerability to administrative barriers of certain dual-eligible subgroups, including racial and ethnic minority groups, who are more likely to face barriers related to structural discrimination at baseline. As states have resumed Medicaid redeterminations, there is a pressing need for policymakers to implement strategies to minimize Medicaid coverage losses, especially for the most vulnerable and minoritized populations.

## Notes

Supplement 1. **eTable.** Summary of Demographic Characteristics, 2015 and 2020 **eFigure 1.** Unadjusted Proportion of Dual-eligible Beneficiaries Regain Medicaid Coverage Within 12 Months, 2015-2019 **eFigure 2.** Adjusted Annual Medicaid Coverage Loss Rate for at Least One Month, By Age Group, 2015-2020 **eFigure 3.** Adjusted Annual Medicaid Coverage Loss Rate for at Least One Month, By Gender, 2015-2020 **eFigure 4.** Adjusted Annual Medicaid Coverage Loss Rate for at Least One Month, By Race and Ethnicity, 2015-2020 **eFigure 5.** Adjusted Annual Medicaid Coverage Loss Rate for at Least One Month, By Original Reason for Medicare Entitlement, 2015-2020 **eFigure 6.** Unadjusted Annual Medicaid Coverage Loss Rate, All Dual-eligible Beneficiaries, 2015-2020 **eFigure 7.** Unadjusted Annual Medicaid Coverage Loss Rate for at Least One Month, By Age Group, 2015-2020 **eFigure 8.** Unadjusted Annual Medicaid Coverage Loss Rate for at Least One Month, By Gender, 2015-2020 **eFigure 9.** Unadjusted Annual Medicaid Coverage Loss Rate for at Least One Month, By Race and Ethnicity, 2015-2020 **eFigure 10.** Unadjusted Annual Medicaid Coverage Loss Rate for at Least One Month, By Dual-eligible Status, 2015-2020 **eFigure 11.** Unadjusted Annual Medicaid Coverage Loss Rate for at Least One Month, By Original Reason for Medicare Entitlement, 2015-2020 **eFigure 12.** Unadjusted Annual Medicaid Coverage Loss Rate for at Least One Month, By TM and MA Plan Type, 2015-2020 **eFigure 13.** Unadjusted Annual Medicaid Coverage Loss Rate, All Dual-Eligible Beneficiaries Irrespective of Part D Low-Income Subsidies, 2015-2020 **eFigure 14.** Relative Likelihood of Dual-eligible Beneficiaries Losing Medicaid Coverage for at Least One Month Between April and December, 2015-2019 vs 2020 **eFigure 15.** Relative Likelihood of Dual-eligible Beneficiaries Losing Medicaid Coverage for at Least One Month, Adjusted for Beneficiary Hierarchical Condition Categories Scores, 2018 **eFigure 16.** Relative Likelihood of Dual-eligible Beneficiaries Losing Medicaid Coverage for at Least One Month, Without Clustering Standard Errors At State Level, 2015-2019 vs 2020

Supplement 2.
Data Sharing Statement
