# Supplementary material for: Medicaid Eligibility Loss Among Dual-Eligible Beneficiaries Before and During COVID-19 Public Health Emergency
Source: JAMA Netw Open. 2024 Apr 11;7(4):e245876. doi: 10.1001/jamanetworkopen.2024.5876 (PMC11009828; doi:10.1001/jamanetworkopen.2024.5876)
Supplement: Supplement 1. — eTable. Summary of Demographic Characteristics, 2015 and 2020 eFigure 1. Unadjusted Proportion of Dual-eligible Beneficiaries Regain Medicaid Coverage Within 12 Months, 2015-2019 eFigure 2. Adjusted Annual Medicaid Coverage Loss Rate for at Least One Month, By Age Group, 2015-2020 eFigure 3. Adjusted Annual Medicaid Coverage Loss Rate for at Least One Month, By Gender, 2015-2020 eFigure 4. Adjusted Annual Medicaid Coverage Loss Rate for at Least One Month, By Race and Ethnicity, 2015-2020 eFigure 5. Adjusted Annual Medicaid Coverage Loss Rate for at Least One Month, By Original Reason for Medicare Entitlement, 2015-2020 eFigure 6. Unadjusted Annual Medicaid Coverage Loss Rate, All Dual-eligible Beneficiaries, 2015-2020 eFigure 7. Unadjusted Annual Medicaid Coverage Loss Rate for at Least One Month, By Age Group, 2015-2020 eFigure 8. Unadjusted Annual Medicaid Coverage Loss Rate for at Least One Month, By Gender, 2015-2020 eFigure 9. Unadjusted Annual Medicaid Coverage Loss Rate for at Least One Month, By Race and Ethnicity, 2015-2020 eFigure 10. Unadjusted Annual Medicaid Coverage Loss Rate for at Least One Month, By Dual-eligible Status, 2015-2020 eFigure 11. Unadjusted Annual Medicaid Coverage Loss Rate for at Least One Month, By Original Reason for Medicare Entitlement, 2015-2020 eFigure 12. Unadjusted Annual Medicaid Coverage Loss Rate for at Least One Month, By TM and MA Plan Type, 2015-2020 eFigure 13. Unadjusted Annual Medicaid Coverage Loss Rate, All Dual-Eligible Beneficiaries Irrespective of Part D Low-Income Subsidies, 2015-2020 eFigure 14. Relative Likelihood of Dual-eligible Beneficiaries Losing Medicaid Coverage for at Least One Month Between April and December, 2015-2019 vs 2020 eFigure 15. Relative Likelihood of Dual-eligible Beneficiaries Losing Medicaid Coverage for at Least One Month, Adjusted for Beneficiary Hierarchical Condition Categories Scores, 2018 eFigure 16. Relative Likelihood of Dual-eligible Beneficiaries Losing Medicaid Coverage for a [file jamanetwopen-e245876-s001.pdf]

Supplemental Online Content

Ma Y, Roberts ET, Johnston K, Orav EJ, Figueroa JF. Medicaid eligibility loss among dual-eligible beneficiaries before and during COVID-19 public health emergency. *JAMA Netw Open.* 2024;7(4):e245876. doi:10.1001/jamanetworkopen.2024.5876

eTable. Summary of Demographic Characteristics, 2015 and 2020

eFigure 1. Unadjusted Proportion of Dual-eligible Beneficiaries Regain Medicaid Coverage Within 12 Months, 2015-2019

eFigure 2. Adjusted Annual Medicaid Coverage Loss Rate for at Least One Month, By Age Group, 2015-2020

eFigure 3. Adjusted Annual Medicaid Coverage Loss Rate for at Least One Month, By Gender, 2015-2020

eFigure 4. Adjusted Annual Medicaid Coverage Loss Rate for at Least One Month, By Race and Ethnicity, 2015-2020

eFigure 5. Adjusted Annual Medicaid Coverage Loss Rate for at Least One Month, By Original Reason for Medicare Entitlement, 2015-2020

eFigure 6. Unadjusted Annual Medicaid Coverage Loss Rate, All Dual-eligible Beneficiaries, 2015-2020

eFigure 7. Unadjusted Annual Medicaid Coverage Loss Rate for at Least One Month, By Age Group, 2015-2020

eFigure 8. Unadjusted Annual Medicaid Coverage Loss Rate for at Least One Month, By Gender, 2015-2020

eFigure 9. Unadjusted Annual Medicaid Coverage Loss Rate for at Least One Month, By Race and Ethnicity, 2015-2020

eFigure 10. Unadjusted Annual Medicaid Coverage Loss Rate for at Least One Month, By Dual-eligible Status, 2015-2020

eFigure 11. Unadjusted Annual Medicaid Coverage Loss Rate for at Least One Month, By Original Reason for Medicare Entitlement, 2015-2020

eFigure 12. Unadjusted Annual Medicaid Coverage Loss Rate for at Least One Month, By TM and MA Plan Type, 2015-2020

© 2024 Ma Y et al. *JAMA Network Open.*

eFigure 13. Unadjusted Annual Medicaid Coverage Loss Rate, All Dual-Eligible Beneficiaries Irrespective of Part D Low-Income Subsidies, 2015-2020

eFigure 14. Relative Likelihood of Dual-eligible Beneficiaries Losing Medicaid Coverage for at Least One Month Between April and December, 2015-2019 vs 2020

eFigure 15. Relative Likelihood of Dual-eligible Beneficiaries Losing Medicaid Coverage for at Least One Month, Adjusted for Beneficiary Hierarchical Condition Categories Scores, 2018

eFigure 16. Relative Likelihood of Dual-eligibles Losing Medicaid Coverage for at Least One Month, Without Clustering Standard Errors At State Level, 2015-2019 vs 2020

This supplemental material has been provided by the authors to give readers additional information about their work.

eTable 1 Summary of Demographic Characteristics, 2015 and 2020

	2015	2020
Age Group		
Less than 55	25.9%	20.9%
55-64	17.4%	18.0%
65-74	28.0%	33.3%
75+	28.7%	27.8%
Gender		
Male	39.6%	40.1%
Female	60.4%	59.9%
Race/Ethnicity		
Non-Hispanic White	55.0%	50.3%
Black	20.6%	20.7%
Hispanic	15.4%	18.8%
Other Race/Ethnicity	8.3%	8.7%
Unknown	0.7%	1.5%
Dual-eligible Status		
Partial Benefit Dual	27.7%	30.2%
Full Benefit Dual	72.3%	69.8%
Original Reason for Medicare Entitlement		
Old age and survivor's insurance (OASI)	43.4%	45.2%
Disability insurance benefits (DIB)	55.3%	53.7%
End-stage renal disease (ESRD)	0.5%	0.6%
Both DIB and ESRD	0.7%	0.5%
Plan Type		
Fee-For-Service	70.3%	54.6%
PACE	0.3%	0.4%
FIDE	1.2%	2.6%
MMP	3.4%	3.6%
Other D-SNPs	12.9%	21.7%
Other conventional, non-integrated MA Plans	12.0%	17.1%

eNotes:

1. Sample limited to beneficiaries who were dually eligible for Medicaid as of January of the year and continuously received low-income subsidies for Medicare Part D prescription drug coverage throughout the year. Beneficiaries who died within a year were excluded from the analyses.
2. Analysis includes beneficiaries in traditional fee-for-service Medicare and beneficiaries in MA plans with prescription drug coverage. Beneficiaries in stand-alone drug plans, employer plans, cost plans, Medicare Savings Account plans, chronic condition special needs plans, or institutional special needs plans are excluded from analyses.

© 2024 Ma Y et al. *JAMA Network Open.*

eFigure 1 Unadjusted Proportion of Dual-eligibles Regain Medicaid Coverage within 12 Months, 2015-2019

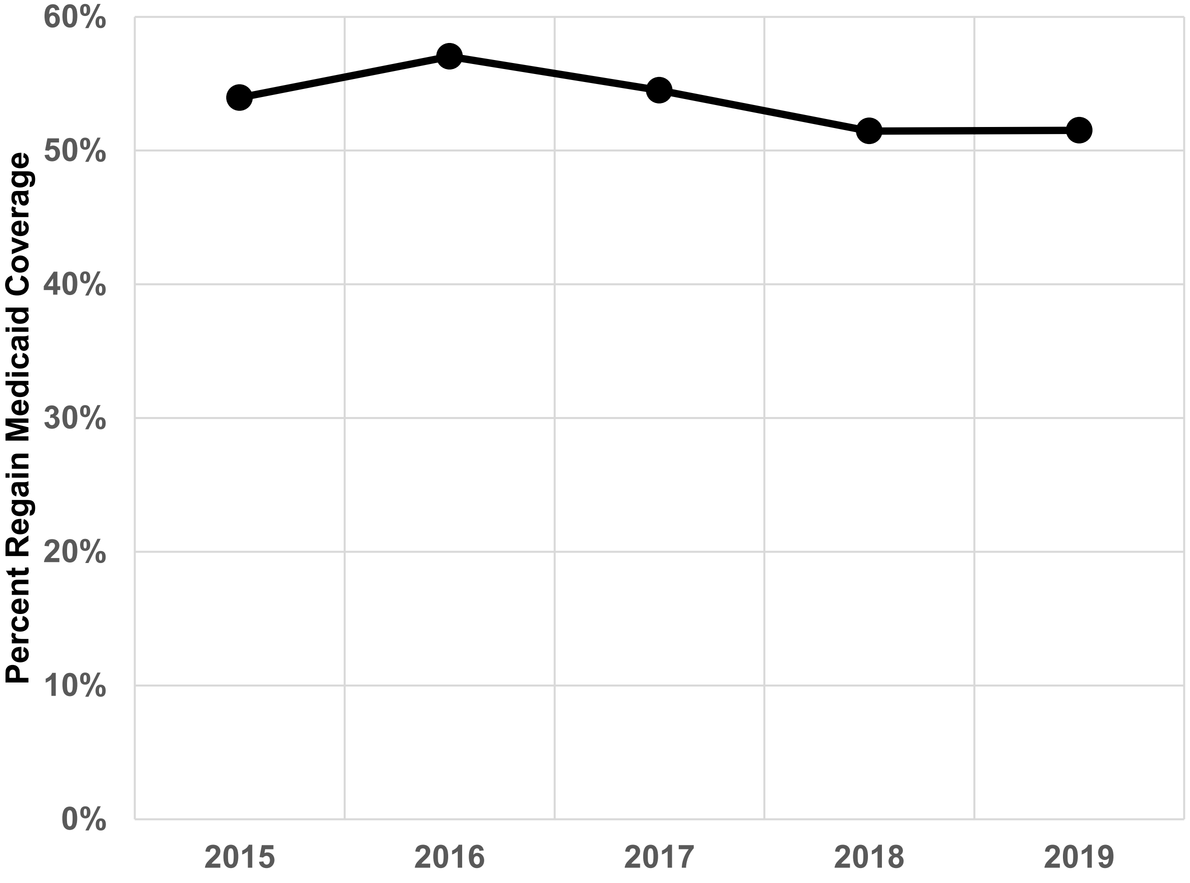

eNotes:

1. Sample limited to beneficiaries who were dually eligible for Medicaid as of January of the year, continuously received low-income subsidies for Medicare Part D prescription drug coverage throughout the year, and lost Medicaid coverage for at least one month in the given year. Beneficiaries who died after losing Medicaid coverage were excluded from the analyses.
2. Percent regaining Medicaid coverage represents the unadjusted proportion of dual-eligibles in the sample who lost Medicaid coverage entirely (i.e., not qualifying for any Medicaid benefits) in a given year and then regained Medicaid coverage within the next 12 months after losing Medicaid coverage.

© 2024 Ma Y et al. *JAMA Network Open.*

eFigure 2 Adjusted Annual Medicaid Coverage Loss Rate for At Least One Month, By Age Group, 2015-2020

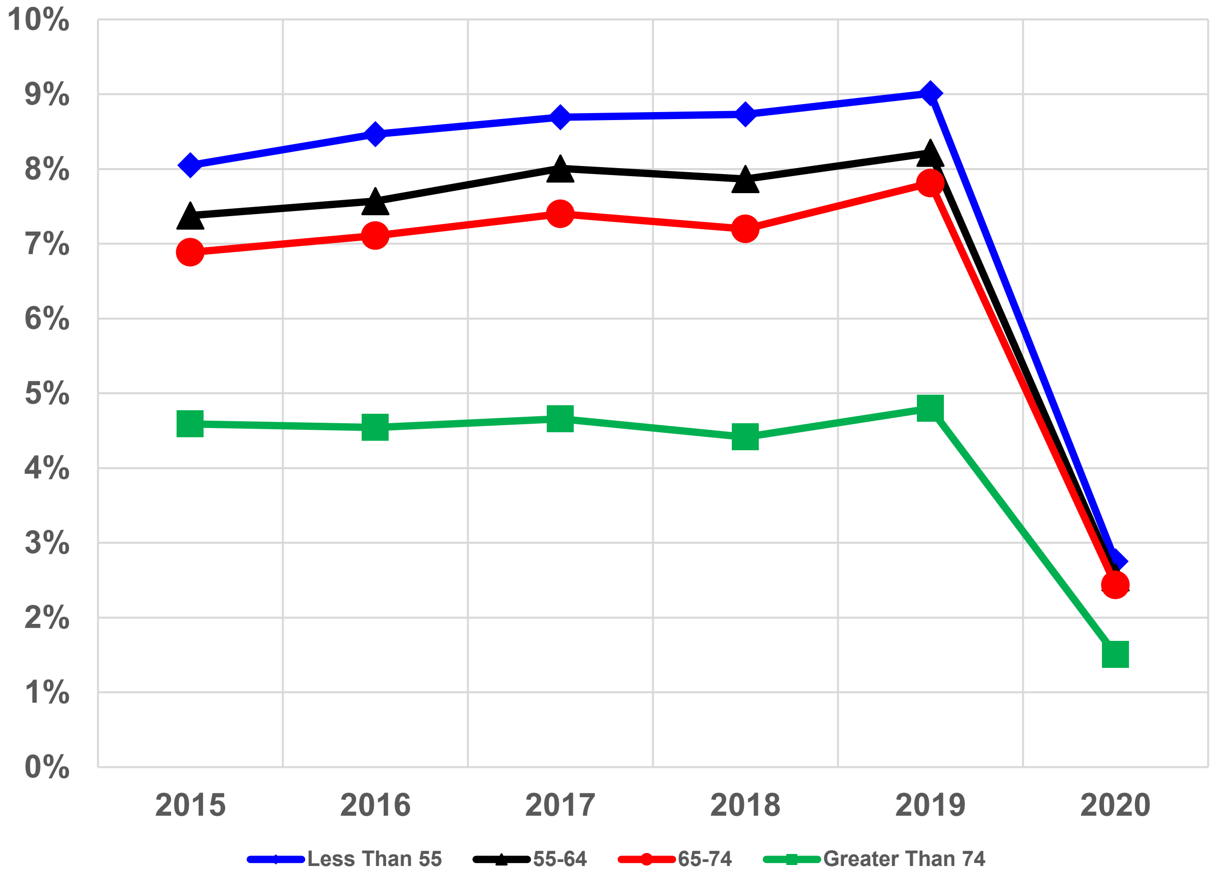

eNotes:

1. Sample limited to beneficiaries who were dually eligible for Medicaid as of January of the year and continuously received low-income subsidies for Medicare Part D prescription drug coverage throughout the year. Beneficiaries who died within a year were excluded from the analyses.
2. Adjusted annual Medicaid coverage loss rate represents the proportion of dual-eligibles in the sample who lost Medicaid coverage entirely (i.e., not qualifying for any Medicaid benefits) in a given year after accounting for beneficiary's age, gender, race/ethnicity, dual-eligible status, original reason for Medicare entitlement, TM and MA plan type, and county fixed effects.

© 2024 Ma Y et al. *JAMA Network Open.*

eFigure 3 Adjusted Annual Medicaid Coverage Loss Rate for At Least One Month, By Gender, 2015-2020

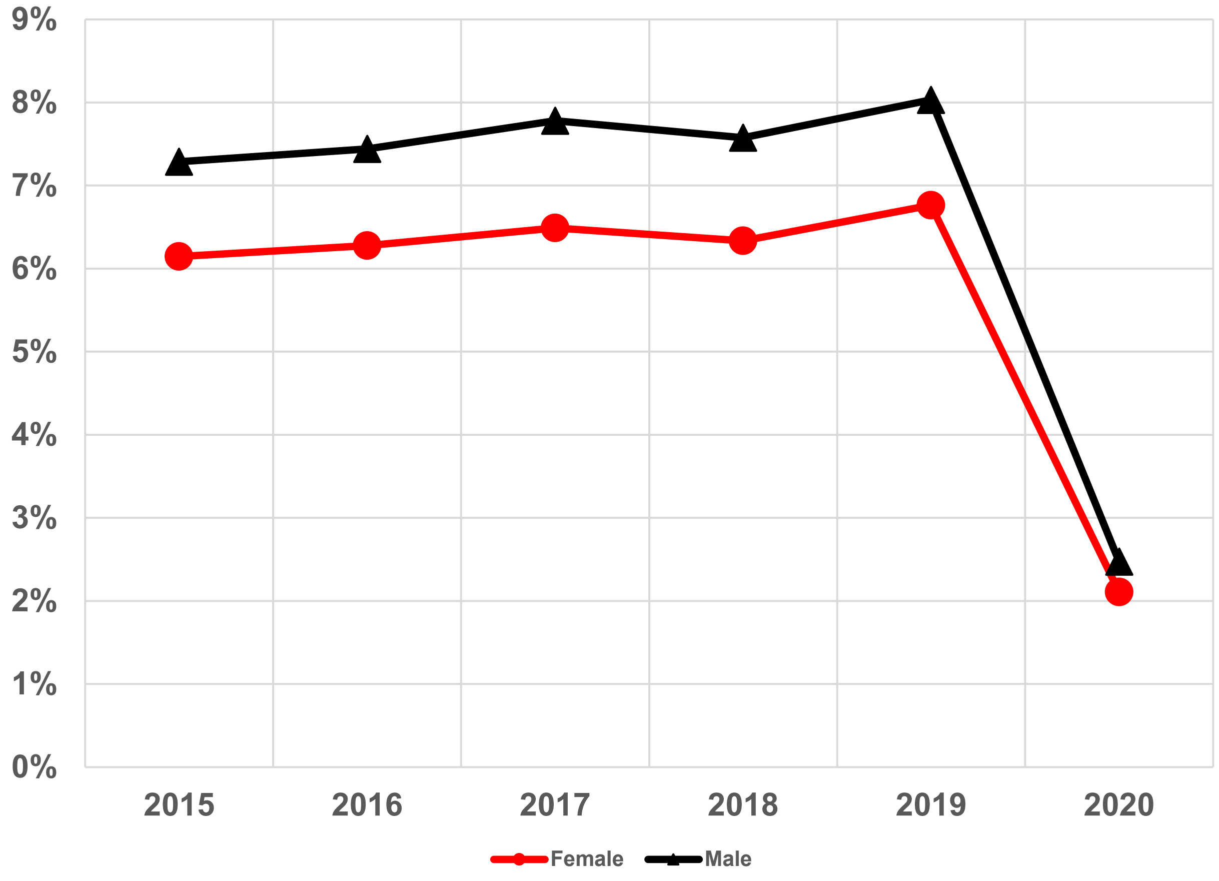

eNotes:

1. Sample limited to beneficiaries who were dually eligible for Medicaid as of January of the year and continuously received low-income subsidies for Medicare Part D prescription drug coverage throughout the year. Beneficiaries who died within a year were excluded from the analyses.
2. Adjusted annual Medicaid coverage loss rate represents the proportion of dual-eligibles in the sample who lost Medicaid coverage entirely (i.e., not qualifying for any Medicaid benefits) in a given year after accounting for beneficiary’s age, gender, race/ethnicity, dual-eligible status, original reason for Medicare entitlement, TM and MA plan type, and county fixed effects.

© 2024 Ma Y et al. *JAMA Network Open.*

eFigure 4 Adjusted Annual Medicaid Coverage Loss Rate for At Least One Month, By Race and Ethnicity, 2015-2020

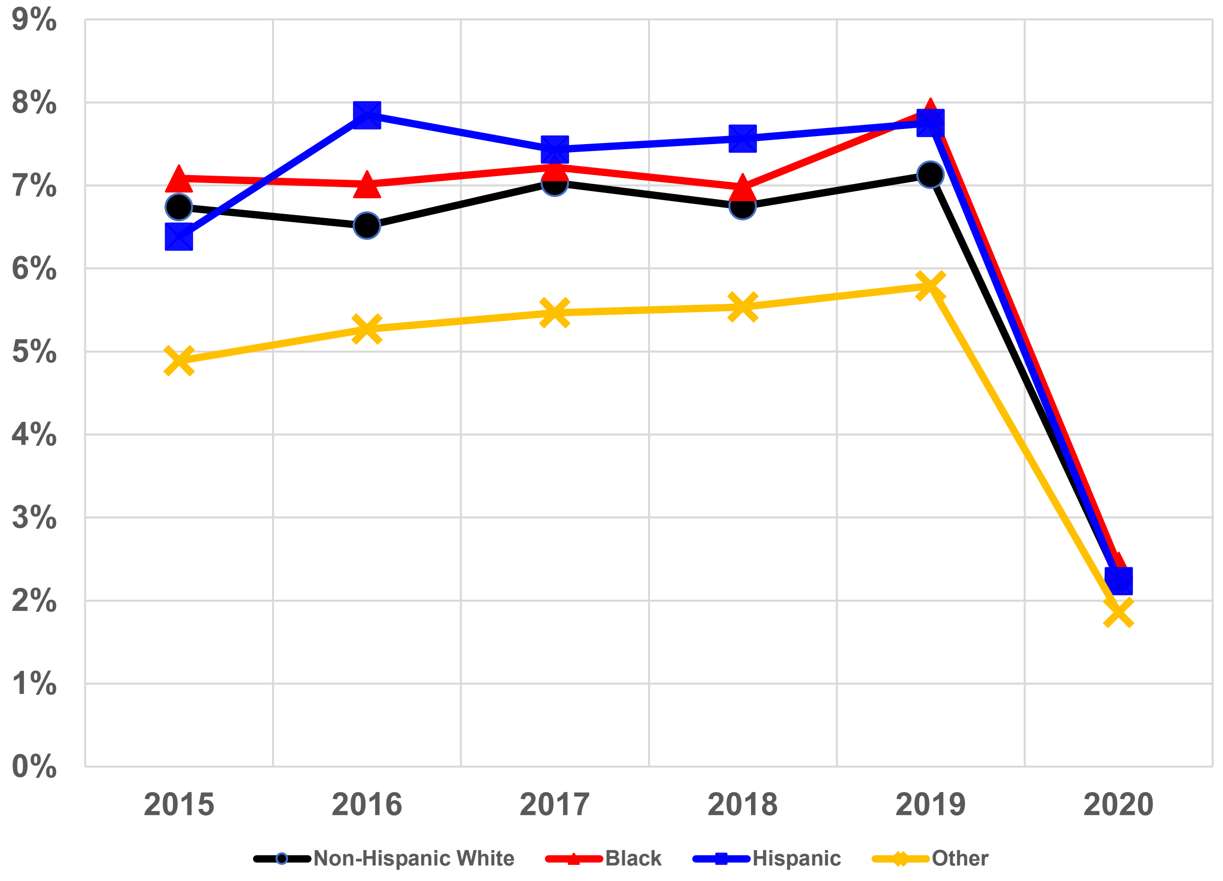

eNotes:

1. Sample limited to beneficiaries who were dually eligible for Medicaid as of January of the year and continuously received low-income subsidies for Medicare Part D prescription drug coverage throughout the year. Beneficiaries who died within a year were excluded from the analyses.
2. Adjusted annual Medicaid coverage loss rate represents the proportion of dual-eligibles in the sample who lost Medicaid coverage entirely (i.e., not qualifying for any Medicaid benefits) in a given year after accounting for beneficiary's age, gender, race/ethnicity, dual-eligible status, original reason for Medicare entitlement, TM and MA plan type, and county fixed effects.
3. Race and ethnicity were defined using the Research Triangle Institute race code. Other race/ethnicity includes Asian/Pacific islander, American Indian/Alaska native, as well as any other race/ethnicity categories that are not non-Hispanic White, Black, or Hispanic.

© 2024 Ma Y et al. *JAMA Network Open.*

eFigure 5 Adjusted Annual Medicaid Coverage Loss Rate for At Least One Month, By Original Reason for Medicare Entitlement, 2015-2020

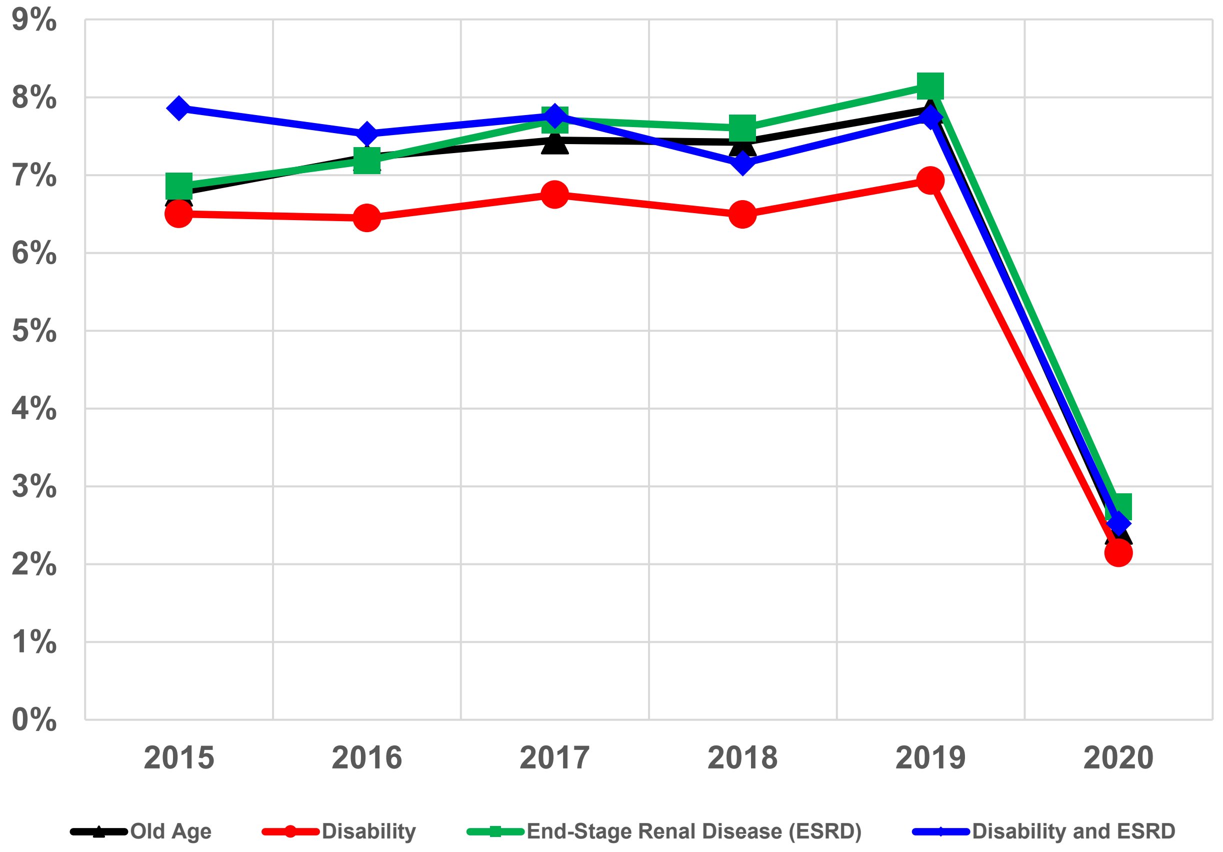

eNotes:

1. Sample limited to beneficiaries who were dually eligible for Medicaid as of January of the year and continuously received low-income subsidies for Medicare Part D prescription drug coverage throughout the year. Beneficiaries who died within a year were excluded from the analyses.
2. Adjusted annual Medicaid coverage loss rate represents the proportion of dual-eligibles in the sample who lost Medicaid coverage entirely (i.e., not qualifying for any Medicaid benefits) in a given year after accounting for beneficiary's age, gender, race/ethnicity, dual-eligible status, original reason for Medicare entitlement, TM and MA plan type, and county fixed effects.

© 2024 Ma Y et al. *JAMA Network Open.*

eFigure 6 Unadjusted Annual Medicaid Coverage Loss Rate, All Dual-eligibles, 2015-2020

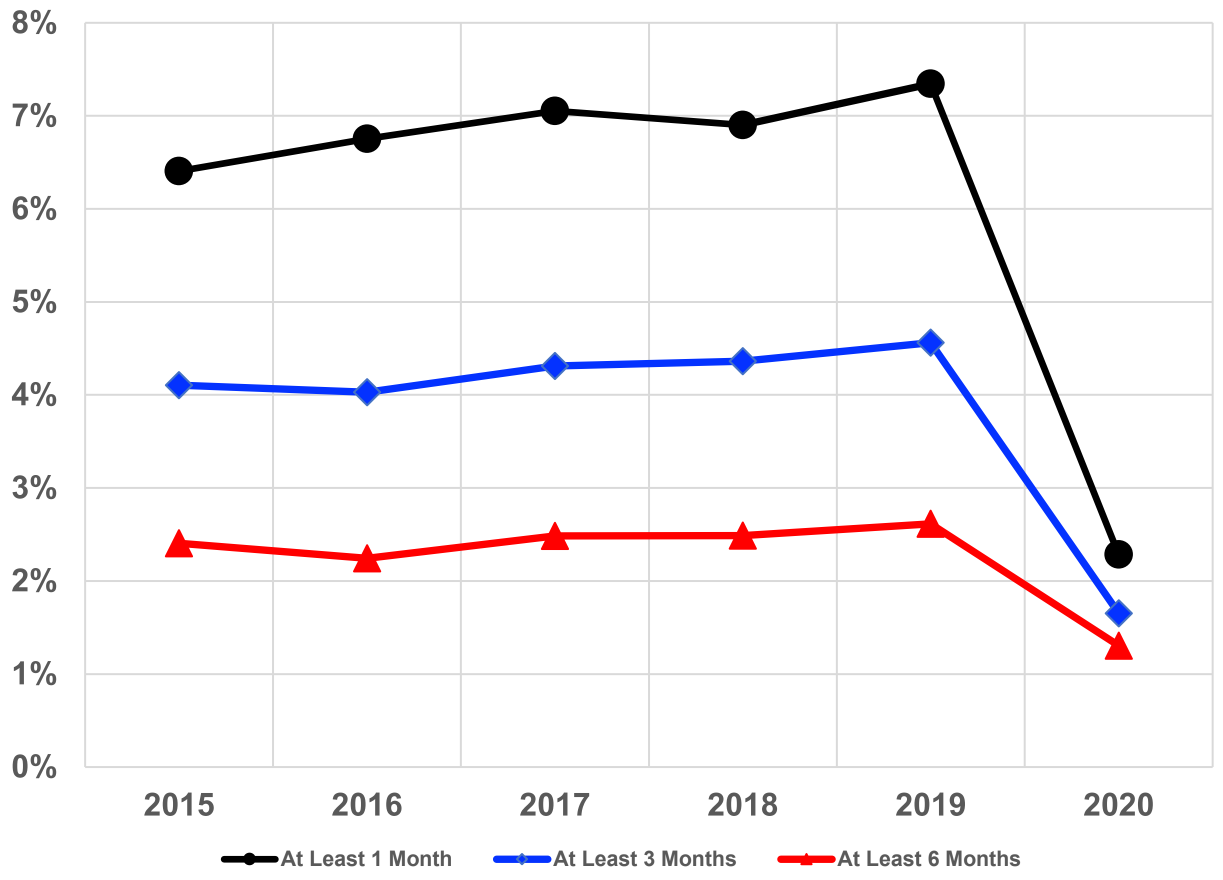

eNotes:

1. Sample limited to beneficiaries who were dually eligible for Medicaid as of January of the year and continuously received low-income subsidies for Medicare Part D prescription drug coverage throughout the year. Beneficiaries who died within a year were excluded from the analyses.
2. Unadjusted annual Medicaid coverage loss rate represents the proportion of dual-eligibles in the sample who lost Medicaid coverage entirely (i.e., not qualifying for any Medicaid benefits) in a given year.

© 2024 Ma Y et al. *JAMA Network Open.*

eFigure 7 Unadjusted Annual Medicaid Coverage Loss Rate for At Least One Month, By Age Group, 2015-2020

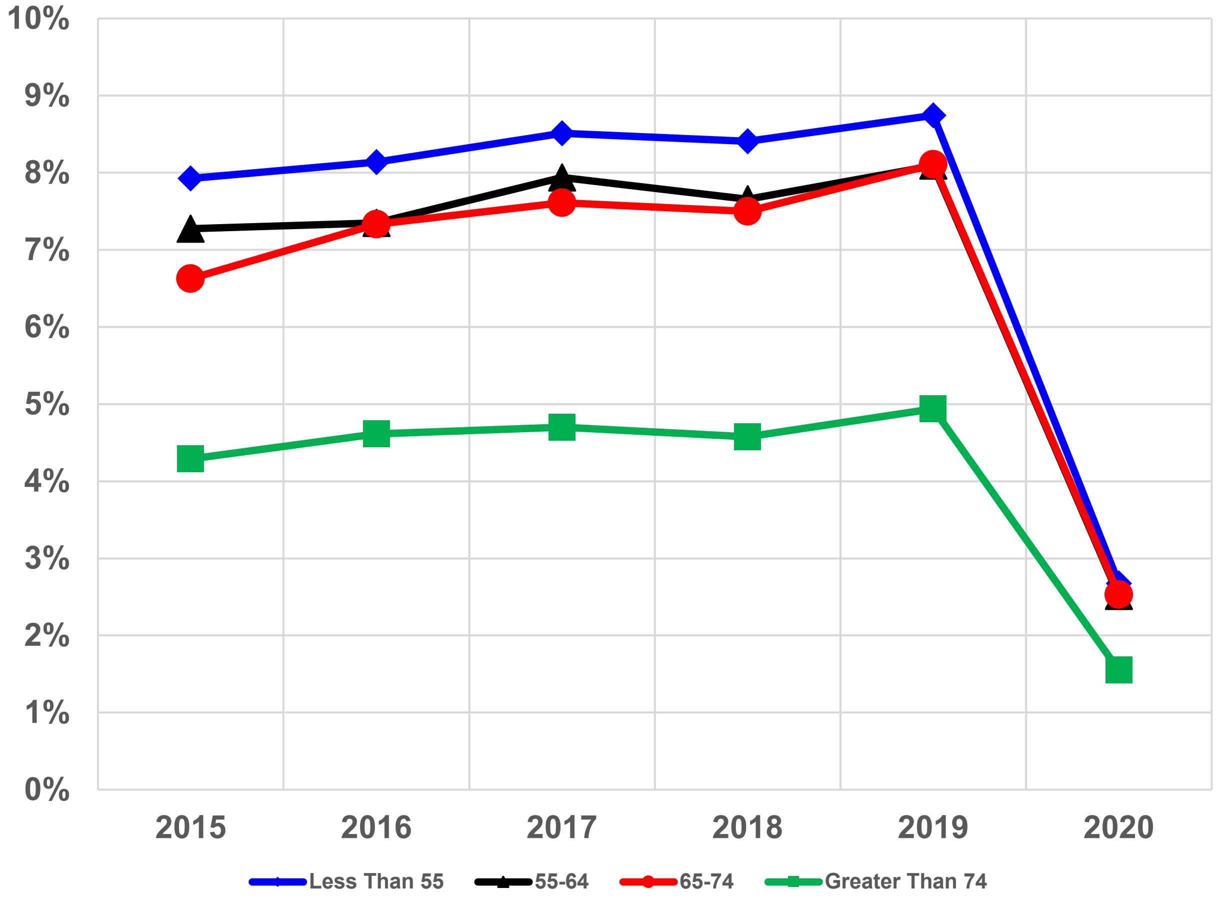

eNotes:

1. Sample limited to beneficiaries who were dually eligible for Medicaid as of January of the year and continuously received low-income subsidies for Medicare Part D prescription drug coverage throughout the year. Beneficiaries who died within a year were excluded from the analyses.
2. Unadjusted annual Medicaid coverage loss rate represents the proportion of dual-eligibles in the sample who lost Medicaid coverage entirely (i.e., not qualifying for any Medicaid benefits) in a given year.

© 2024 Ma Y et al. *JAMA Network Open.*

eFigure 8 Unadjusted Annual Medicaid Coverage Loss Rate for At Least One Month, By Gender, 2015-2020

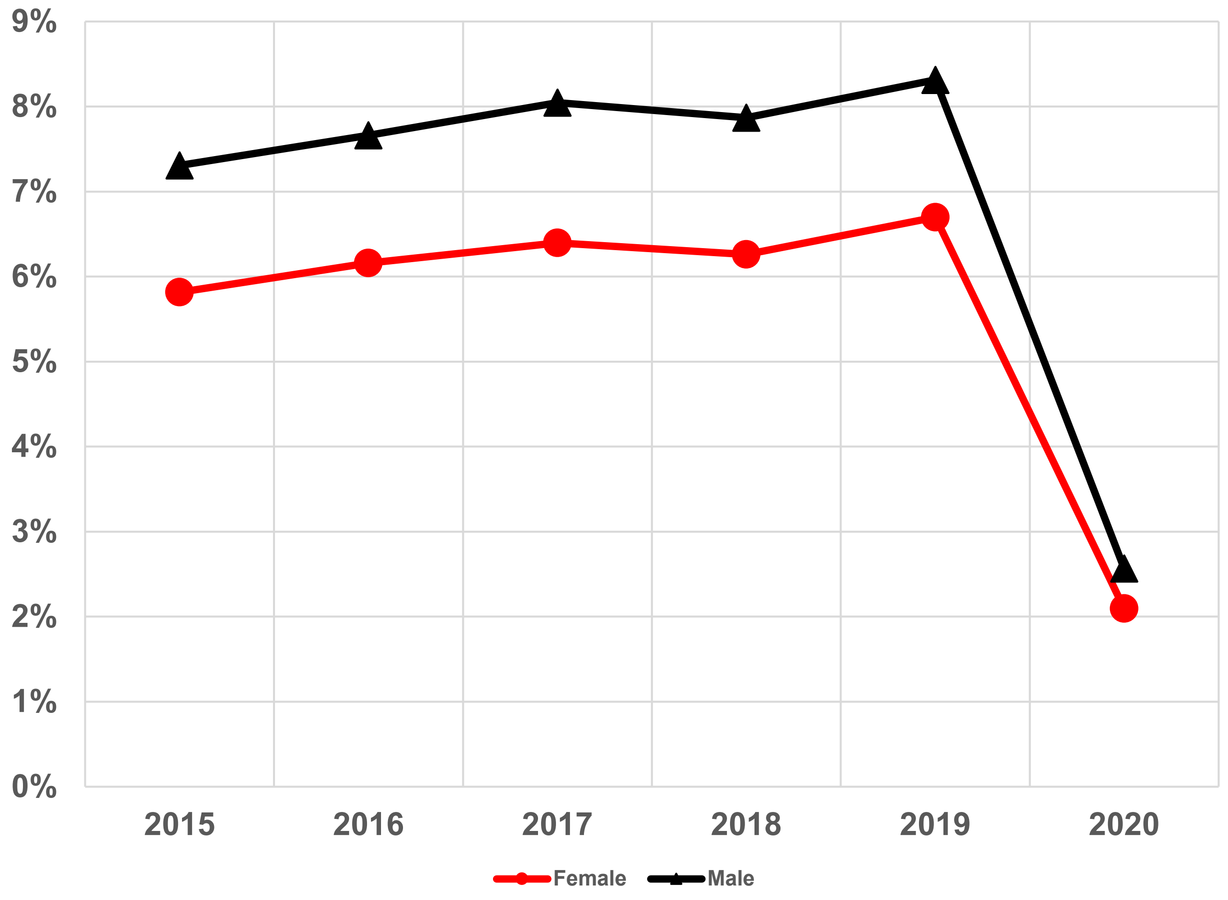

eNotes:

1. Sample limited to beneficiaries who were dually eligible for Medicaid as of January of the year and continuously received low-income subsidies for Medicare Part D prescription drug coverage throughout the year. Beneficiaries who died within a year were excluded from the analyses.
2. Unadjusted annual Medicaid coverage loss rate represents the proportion of dual-eligibles in the sample who lost Medicaid coverage entirely (i.e., not qualifying for any Medicaid benefits) in a given year.

© 2024 Ma Y et al. *JAMA Network Open.*

eFigure 9 Unadjusted Annual Medicaid Coverage Loss Rate for At Least One Month, By Race and Ethnicity, 2015-2020

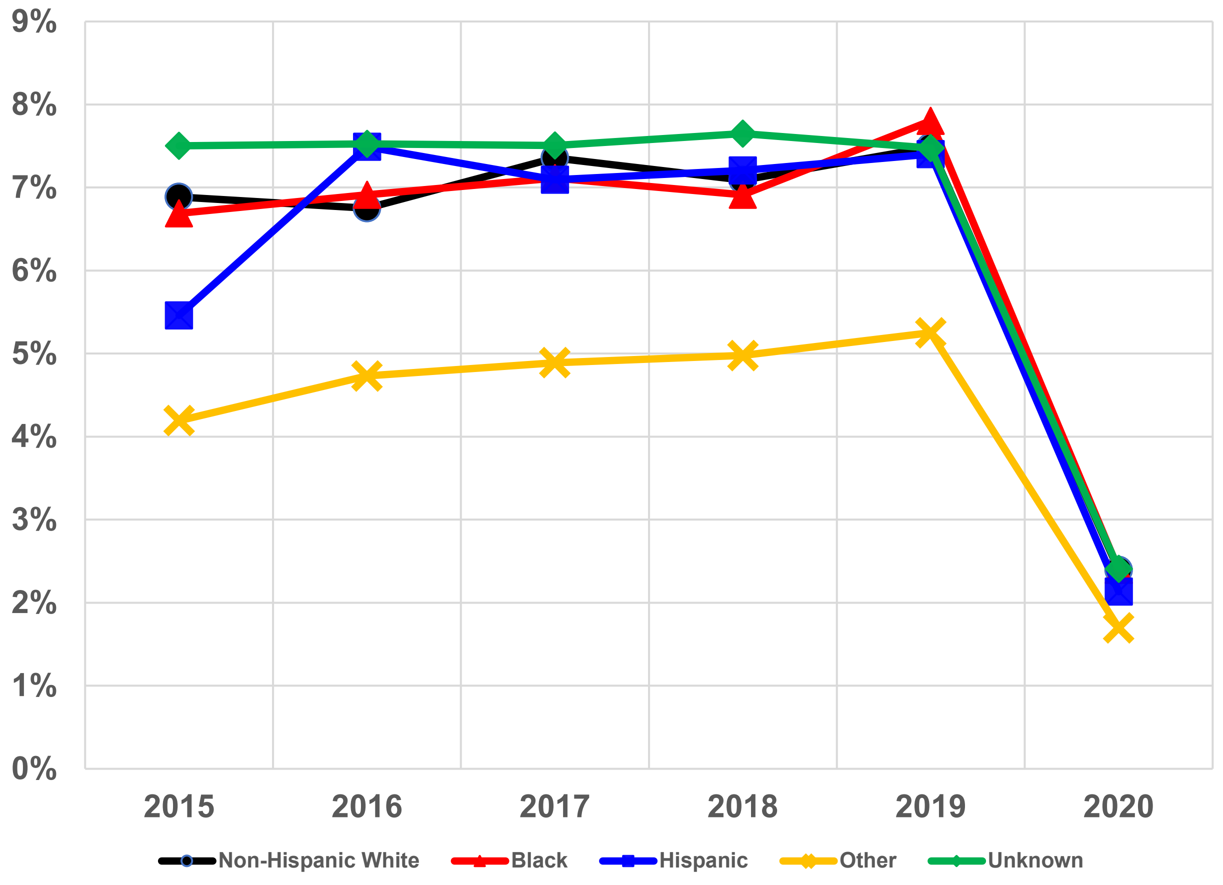

eNotes:

1. Sample limited to beneficiaries who were dually eligible for Medicaid as of January of the year and continuously received low-income subsidies for Medicare Part D prescription drug coverage throughout the year. Beneficiaries who died within a year were excluded from the analyses.
2. Unadjusted annual Medicaid coverage loss rate represents the proportion of dual-eligibles in the sample who lost Medicaid coverage entirely (i.e., not qualifying for any Medicaid benefits) in a given year.
3. Race and ethnicity were defined using the Research Triangle Institute race code. Other race/ethnicity includes Asian/Pacific islander, American Indian/Alaska native, as well as any other race/ethnicity categories that are not non-Hispanic White, Black, or Hispanic.

© 2024 Ma Y et al. *JAMA Network Open.*

eFigure 10 Unadjusted Annual Medicaid Coverage Loss Rate for At Least One Month, By Dual-eligible Status, 2015-2020

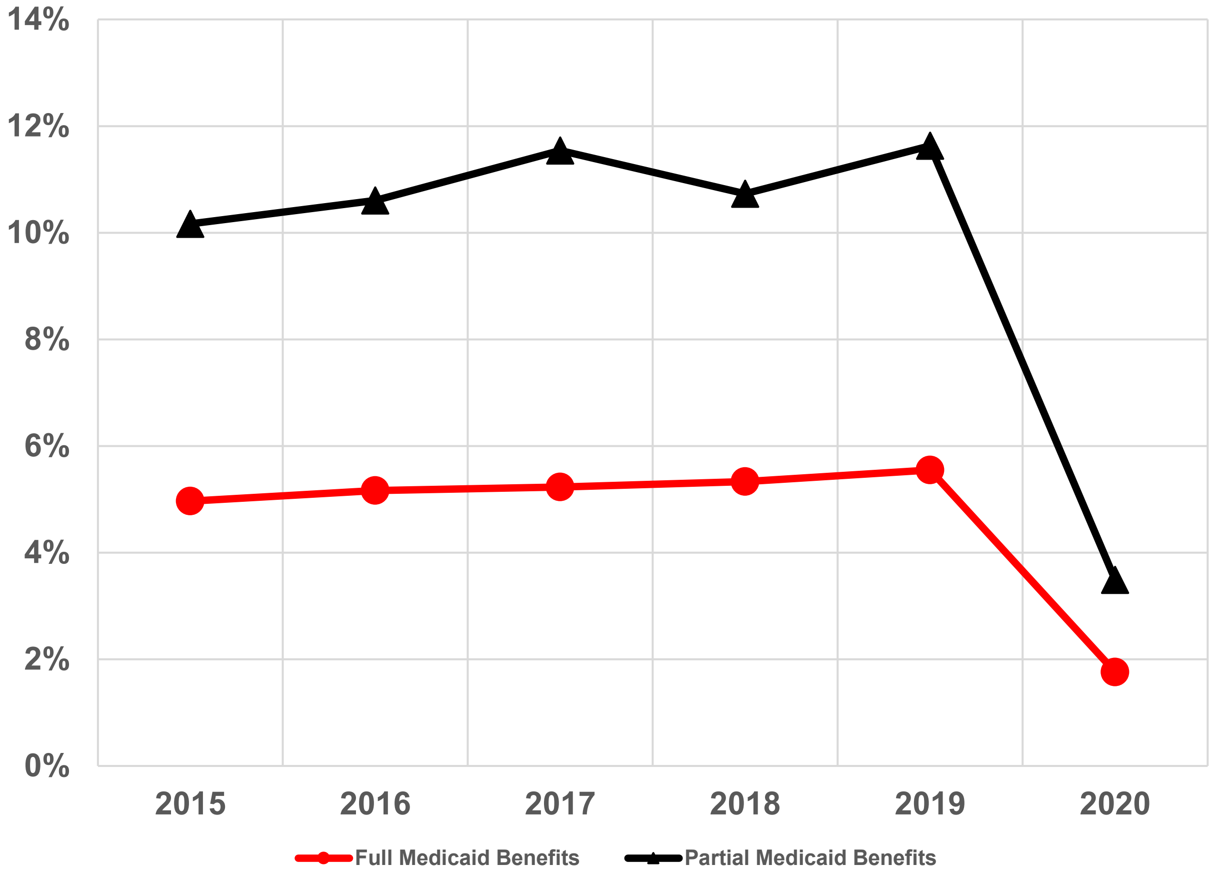

eNotes:

1. Sample limited to beneficiaries who were dually eligible for Medicaid as of January of the year and continuously received low-income subsidies for Medicare Part D prescription drug coverage throughout the year. Beneficiaries who died within a year were excluded from the analyses.
2. Unadjusted annual Medicaid coverage loss rate represents the proportion of dual-eligibles in the sample who lost Medicaid coverage entirely (i.e., not qualifying for any Medicaid benefits) in a given year.
3. Full Medicaid benefits dual-eligibles include Qualified Medicare Beneficiary (QMB) with full Medicaid coverage, Specified Low-Income Medicare Beneficiary (SLMB) with full Medicaid coverage, and other dual eligibles with full Medicaid coverage. Partial Medicaid benefits dual-eligibles include QMB-only, SLMB-only, Qualified Disabled Working Individual, and Qualifying Individuals.

© 2024 Ma Y et al. *JAMA Network Open.*

eFigure 11 Unadjusted Annual Medicaid Coverage Loss Rate for At Least One Month, By Original Reason for Medicare Entitlement, 2015-2020

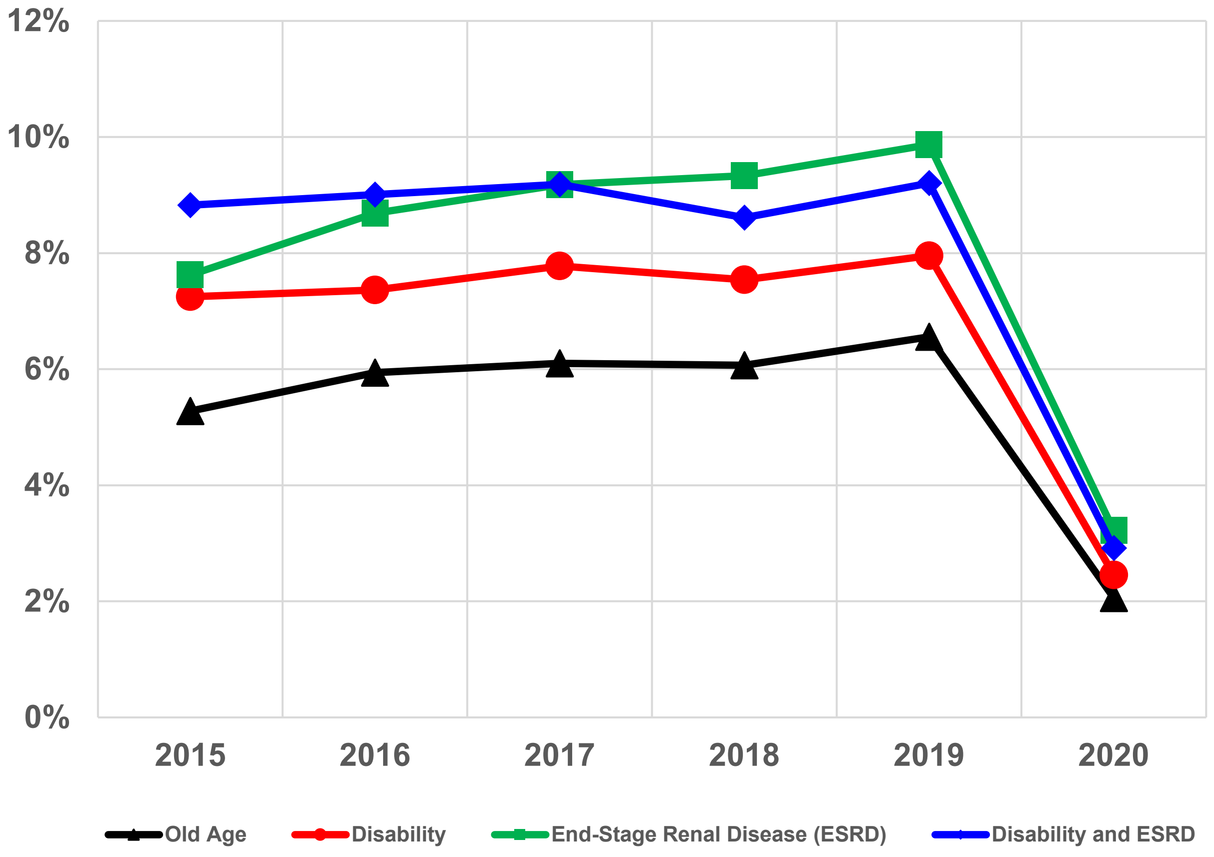

eNotes:

1. Sample limited to beneficiaries who were dually eligible for Medicaid as of January of the year and continuously received low-income subsidies for Medicare Part D prescription drug coverage throughout the year. Beneficiaries who died within a year were excluded from the analyses.
2. Unadjusted annual Medicaid coverage loss rate represents the proportion of dual-eligibles in the sample who lost Medicaid coverage entirely (i.e., not qualifying for any Medicaid benefits) in a given year.

© 2024 Ma Y et al. *JAMA Network Open.*

eFigure 12 Unadjusted Annual Medicaid Coverage Loss Rate for At Least One Month, By TM and MA Plan Type, 2015-2020

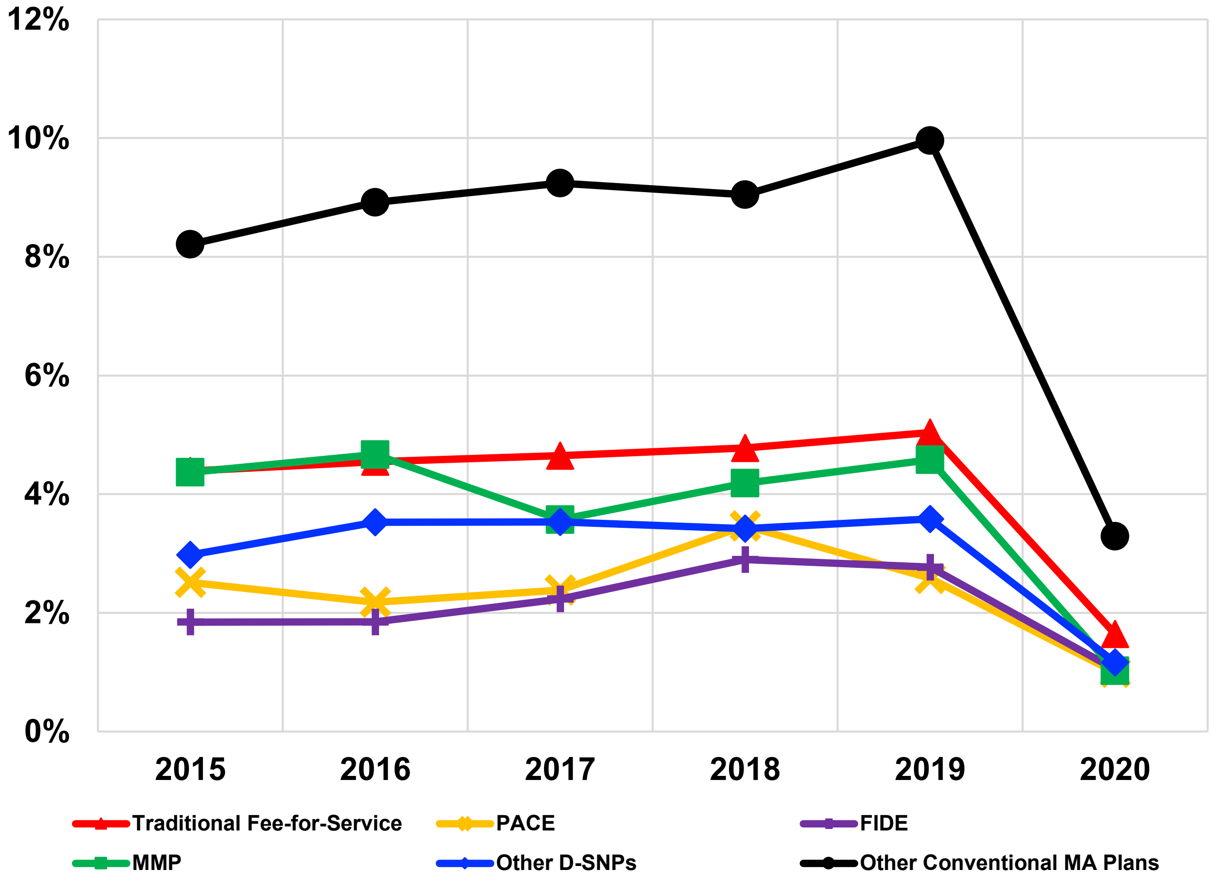

eNotes:

1. Sample limited to beneficiaries who were dually eligible for Medicaid as of January of the year and continuously received low-income subsidies for Medicare Part D prescription drug coverage throughout the year. Beneficiaries who died within a year were excluded from the analyses. For comparability across different TM and MA plan types, sample was further limited to dual-eligibles aged 55 or above with full Medicaid benefits.
2. Unadjusted annual Medicaid coverage loss rate represents the proportion of dual-eligibles in the sample who lost Medicaid coverage entirely (i.e., not qualifying for any Medicaid benefits) in a given year.
3. Analysis includes beneficiaries in traditional fee-for-service Medicare and beneficiaries in MA plans with prescription drug coverage. Beneficiaries in stand-alone drug plans, employer plans, cost plans, Medicare Savings Account plans, chronic condition special needs plans, or institutional special needs plans are excluded from analyses.

© 2024 Ma Y et al. *JAMA Network Open.*

eFigure 13 Unadjusted Annual Medicaid Coverage Loss Rate, All Dual-Eligibles Irrespective of Part D Low-Income Subsidies, 2015-2020

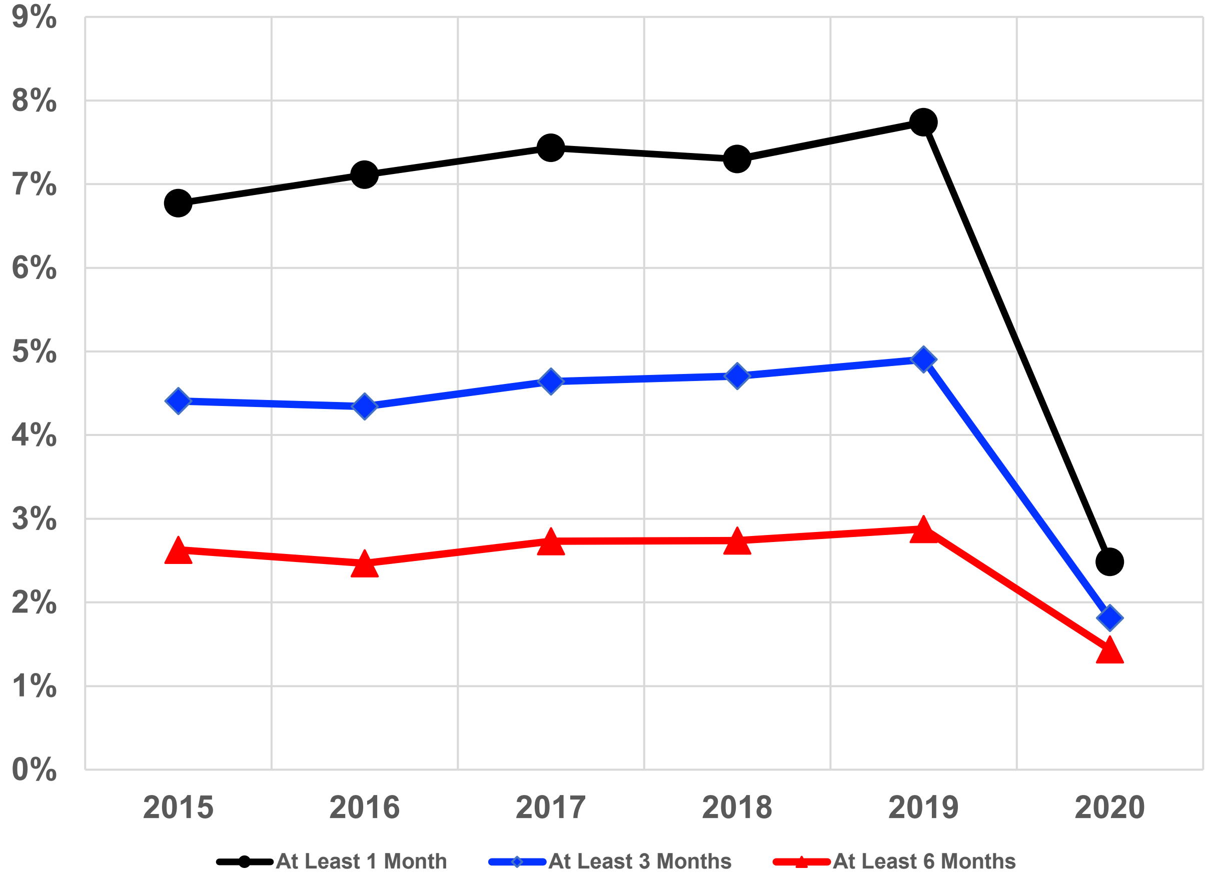

Notes:

1. Sample limited to beneficiaries who were dually eligible for Medicaid as of January of the year.
2. Unadjusted annual Medicaid coverage loss rate represents the proportion of dual-eligibles in the sample who lost Medicaid coverage entirely (i.e., not qualifying for any Medicaid benefits) in a given year.

© 2024 Ma Y et al. *JAMA Network Open.*

eFigure 14 Relative Likelihood of Dual-eligibles Losing Medicaid Coverage for At Least One Month between April and December, 2015-2019 vs. 2020

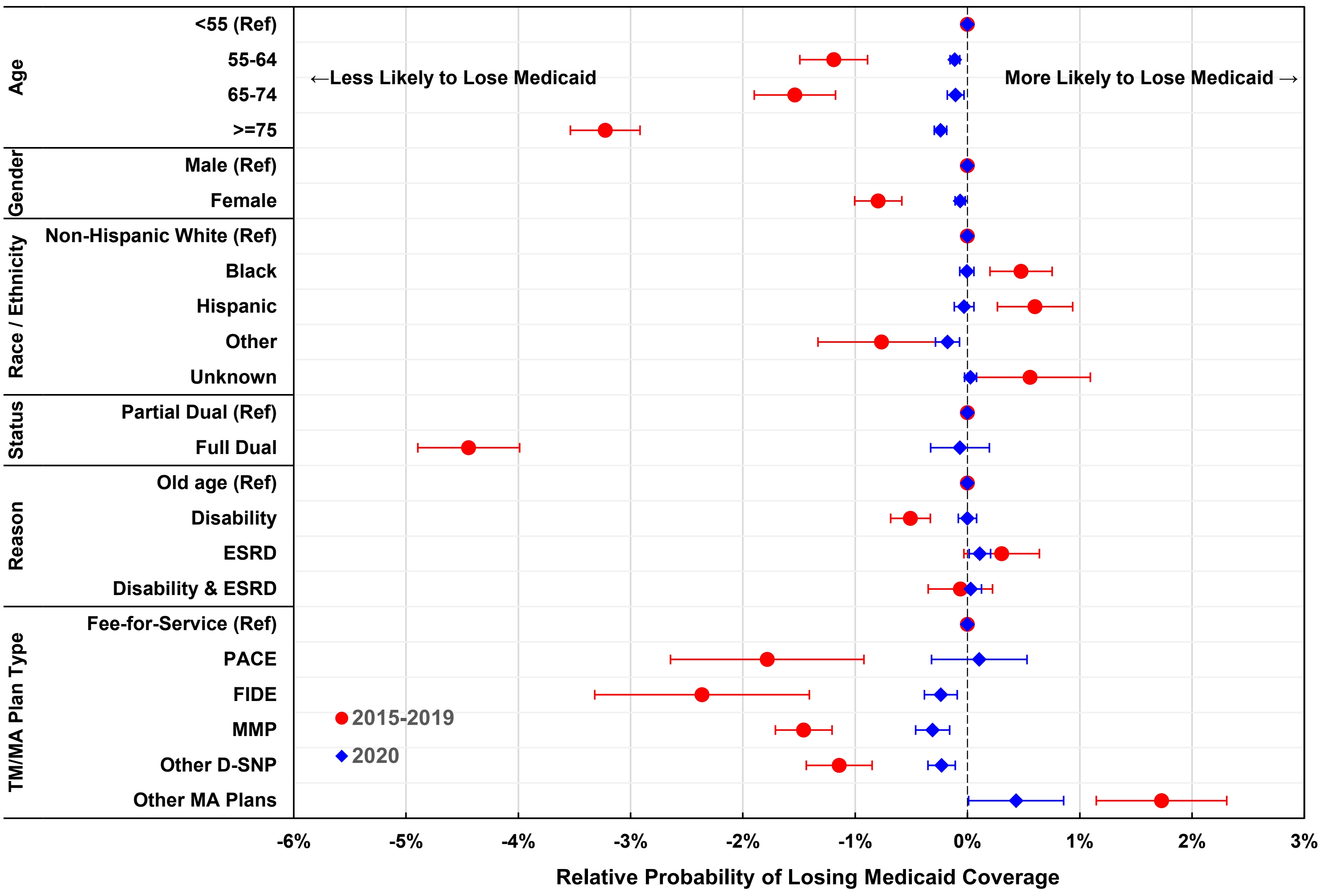

© 2024 Ma Y et al. *JAMA Network Open.*

eNotes:

1. Sample limited to beneficiaries who were dually eligible for Medicaid as of April of the year and continuously received low-income subsidies for Medicare Part D prescription drug coverage throughout rest of the year. Beneficiaries who died within a year were excluded from the analyses.
2. Analysis includes beneficiaries in traditional fee-for-service Medicare and beneficiaries in MA plans with prescription drug coverage. Beneficiaries in stand-alone drug plans, employer plans, cost plans, Medicare Savings Account plans, chronic condition special needs plans, or institutional special needs plans are excluded from analyses.
3. Relative likelihood of dual-eligibles losing Medicaid coverage is estimated from logistic regression model accounting for beneficiary's age, gender, race/ethnicity, dual-eligible status, original reason for Medicare entitlement, TM and MA plan type, and county fixed effects. It represents the average difference in probability of losing Medicaid coverage compared to the reference group.
4. Race and ethnicity were defined using the Research Triangle Institute race code. Other race/ethnicity includes Asian/Pacific islander, American Indian/Alaska native, as well as any other race/ethnicity categories that are not non-Hispanic White, Black, or Hispanic.

© 2024 Ma Y et al. *JAMA Network Open.*

eFigure 15 Relative Likelihood of Dual-eligibles Losing Medicaid Coverage for At Least One Month, Adjusted for Beneficiary Hierarchical Condition Categories Scores, 2018

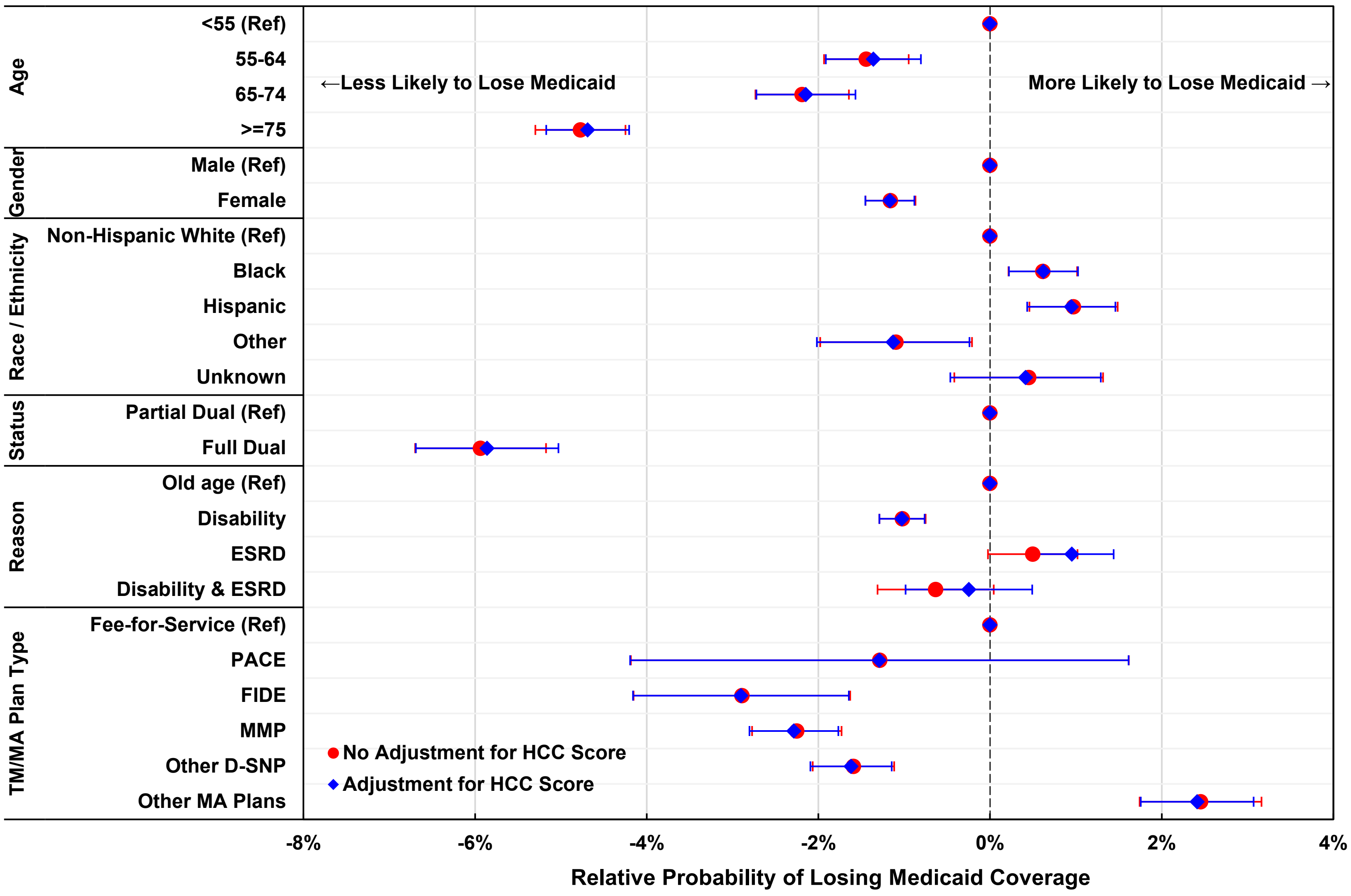

© 2024 Ma Y et al. *JAMA Network Open.*

eNotes:

1. Sample limited to beneficiaries who were dually eligible for Medicaid as of January of 2018 and continuously received low-income subsidies for Medicare Part D prescription drug coverage throughout 2018. Beneficiaries who died within 2018 were excluded from the analyses.
2. Analyses limited to 20% sample where we have TM claims data and MA encounter data available.
3. Analysis includes beneficiaries in traditional fee-for-service Medicare and beneficiaries in MA plans with prescription drug coverage. Beneficiaries in stand-alone drug plans, employer plans, cost plans, Medicare Savings Account plans, chronic condition special needs plans, or institutional special needs plans are excluded from analyses.
4. Relative likelihood of dual-eligibles losing Medicaid coverage is estimated from logistic regression model accounting for beneficiary's age, gender, race/ethnicity, dual-eligible status, original reason for Medicare entitlement, TM and MA plan type, and county fixed effects. It represents the average difference in probability of losing Medicaid coverage compared to the reference group.
5. Race and ethnicity were defined using the Research Triangle Institute race code. Other race/ethnicity includes Asian/Pacific islander, American Indian/Alaska native, as well as any other race/ethnicity categories that are not non-Hispanic White, Black, or Hispanic.
6. Given known issues of more aggressive coding in MA, we deflated 2018 MA HCC scores by 6% following prior studies.

© 2024 Ma Y et al. *JAMA Network Open.*

eFigure 16 Relative Likelihood of Dual-eligibles Losing Medicaid Coverage for At Least One Month, Without Clustering Standard Errors At State Level, 2015-2019 vs. 2020

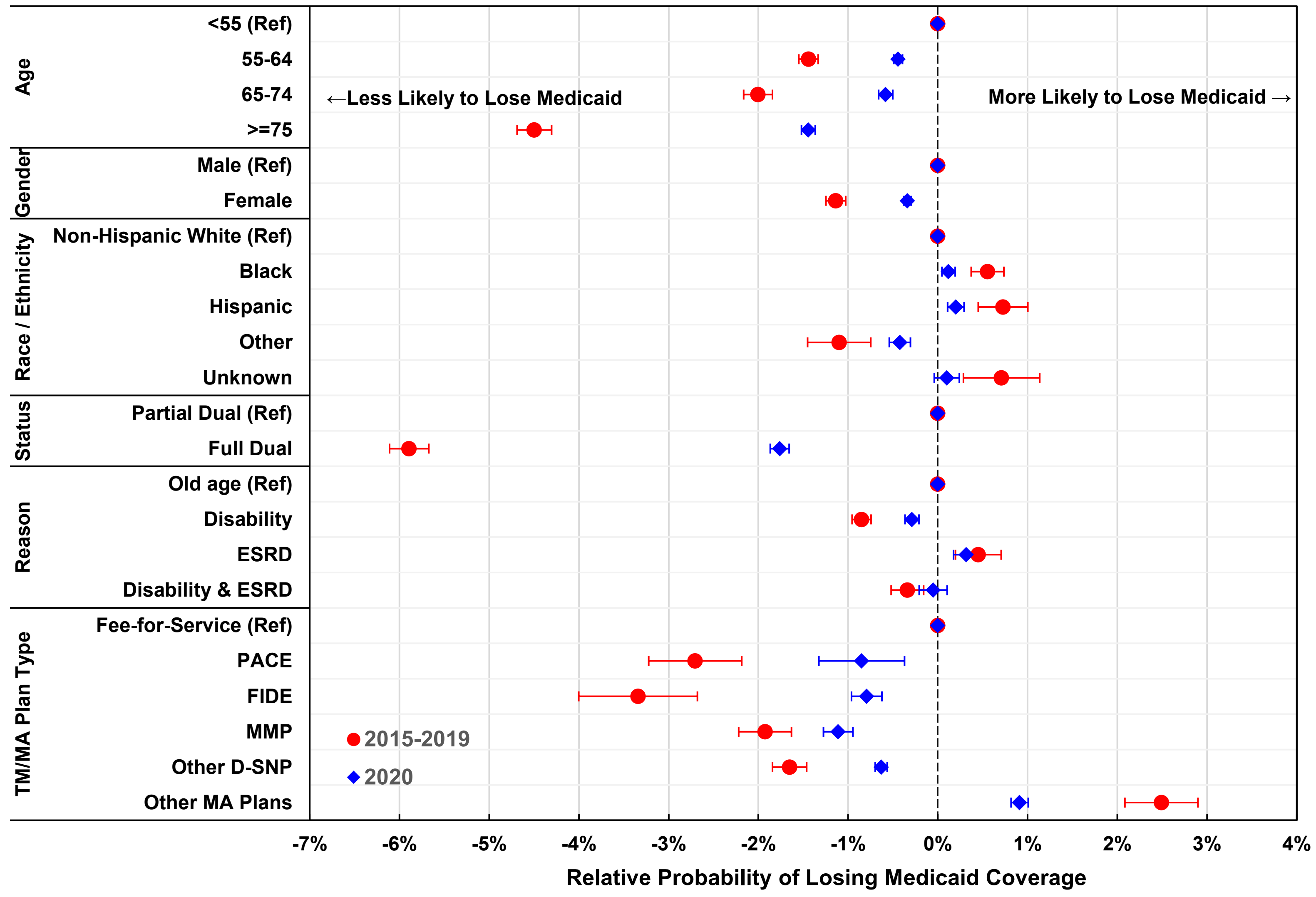

© 2024 Ma Y et al. *JAMA Network Open.*

eNotes:

1. Sample limited to beneficiaries who were dually eligible for Medicaid as of January of the year and continuously received low-income subsidies for Medicare Part D prescription drug coverage throughout the year. Beneficiaries who died within a year were excluded from the analyses.
2. Analysis includes beneficiaries in traditional fee-for-service Medicare and beneficiaries in MA plans with prescription drug coverage. Beneficiaries in stand-alone drug plans, employer plans, cost plans, Medicare Savings Account plans, chronic condition special needs plans, or institutional special needs plans are excluded from analyses.
3. Relative likelihood of dual-eligibles losing Medicaid coverage is estimated from logistic regression model accounting for beneficiary's age, gender, race/ethnicity, dual-eligible status, original reason for Medicare entitlement, TM and MA plan type, and county fixed effects without clustering standard errors at the state level. It represents the average difference in probability of losing Medicaid coverage compared to the reference group.
4. Race and ethnicity were defined using the Research Triangle Institute race code. Other race/ethnicity includes Asian/Pacific islander, American Indian/Alaska native, as well as any other race/ethnicity categories that are not non-Hispanic White, Black, or Hispanic.

© 2024 Ma Y et al. *JAMA Network Open.*
