# Supplementary material for: Medicaid Eligibility Loss Among Dual-Eligible Beneficiaries Before and During COVID-19 Public Health Emergency
Source: JAMA Netw Open. 2024 Apr 11;7(4):e245876. doi: 10.1001/jamanetworkopen.2024.5876 (PMC11009828; doi:10.1001/jamanetworkopen.2024.5876)
Supplement: Supplement 2. — Data Sharing Statement [file jamanetwopen-e245876-s002.pdf]

Data Sharing Statement

Data

Data available: No

Additional Information

Explanation for why data not available: The data is restricted given our DUA with federal government and cannot be shared. However, any researcher can apply directly for these data and replicate study using methods.
